# Supplementary material for: Vitamin C-induced CO2 capture enables high-rate ethylene production in CO2 electroreduction
Source: Nat Commun. 2024 Jan 2;15:192. doi: 10.1038/s41467-023-44586-0 (PMC10762245; doi:10.1038/s41467-023-44586-0)
Supplement: Supplementary file 1 — Supplementary Information [file 41467_2023_44586_MOESM1_ESM.pdf]

Supplementary Information

Vitamin C-induced CO_2 capture enables high-rate ethylene production in CO_2 electroreduction

Jongyoun Kim[1,†], Taemin Lee[1,†], Hyun Dong Jung[2,†], Minkyoung Kim[1], Jungsu Eo[1], Byeongjae Kang[1], Hyeonwoo Jung[1], Jaehyoung Park[1], Daewon Bae[1], Yujin Lee[1], Sojung Park[3], Wooyul Kim[3], Seoin Back[2,*], Youngu Lee[1,*] and Dae-Hyun Nam[1,*]

[1]Department of Energy Science and Engineering, Daegu Gyeongbuk Institute of Science and Technology (DGIST), Daegu, 42988, Republic of Korea

[2]Department of Chemical and Biomolecular Engineering, Institute of Emergent Materials, Sogang University, Seoul, 04107, Republic of Korea

[3]Department of Energy Engineering, Institute for Environmental and Climate Technology, Korea Institute of Energy Technology (KENTECH), Naju, Jeollanam-do, 58330, Republic of Korea

[†]These authors contributed equally to this paper.

*Corresponding author:

Dae-Hyun Nam, e-mail: dhnam@dgist.ac.kr

Youngu Lee, e-mail: youngulee@dgist.ac.kr

Seoin Back, e-mail: sback@sogang.ac.kr

Modeling of Electrochemical CO_2 Reduction

To simulate the CO_2 reduction reaction (CO_2RR) mechanism, we initiated the process with CO_2 adsorption, followed by two protonation steps to form *COOH and *CO. Subsequently, we examined the *CO dimerization, which is reported as the rate-determining step (RDS) for the production of ethylene.[1, 2] Also, the competing hydrogen evolution reaction (HER) was considered.

(R1a, R2a, R3a, R4a, R5a, R6a) and (R1b, R2b, R2c, R3b, R4b, R5b, R5c, R6b, R6c) represent the modeled reactions for Cu (100) and AA/Cu (100), respectively. Using the liquid configuration obtained from the AIMD simulations, we calculated the reaction Gibbs energies for the key reactions. Note that the initial and final states have an identical number of atoms in all cases except for the initial CO_2 adsorption step (1) and the second step of HER (6). The energy of the initial state of CO_2 adsorption was calculated as the sum of energies of the bare surface and gaseous CO_2 molecule and the energy of the final state of H_2 production was calculated as the sum of energies of the bare surface and gaseous H_2 molecule. In the following reaction expressions, * denotes the catalyst surface.

(1) CO_2 adsorption

$$* + \mathrm{CO_2}\ (\mathrm{g}) \rightarrow * \mathrm{CO_2} \qquad \text{(R1a)}$$

$$* + \mathrm{AA} + \mathrm{CO_2}\ (\mathrm{g}) \rightarrow * \mathrm{CO_2} + \mathrm{AA} \qquad \text{(R1b)}$$

(2) Protonation of $*CO_2$

$$* \mathrm{CO_2} + \mathrm{H_2O} + \mathrm{e^-} \rightarrow * \mathrm{COOH} + \mathrm{OH^-} \qquad \text{(R2a)}$$

$$* \mathrm{CO_2} + \mathrm{H_2O} + \mathrm{AA} + \mathrm{e^-} \rightarrow * \mathrm{COOH} + \mathrm{OH^-} + \mathrm{AA} \qquad \text{(R2b)}$$

$$* \mathrm{CO_2} + \mathrm{H_2O} + \mathrm{AA} + \mathrm{e^-} \rightarrow * \mathrm{COOH} + \mathrm{H_2O} + \mathrm{ASC^-} \qquad \text{(R2c)}$$

(3) Protonation of *COOH

$$* \mathrm{COOH} + \mathrm{H_2O} + \mathrm{e^-} \rightarrow * \mathrm{CO} + \mathrm{H_2O} + \mathrm{OH^-} \quad \text{(R3a)}$$

$$* \mathrm{COOH} + \mathrm{H_2O} + \mathrm{AA} + \mathrm{e^-} \rightarrow * \mathrm{CO} + 2\mathrm{H_2O} + \mathrm{ASC^-} \quad \text{(R3b)}$$

(4) *CO dimerization

$$* 2\mathrm{CO} \rightarrow * \mathrm{OCCO} \quad \text{(R4a)}$$

$$* 2\mathrm{CO} + \mathrm{AA} \rightarrow * \mathrm{OCCO} + \mathrm{AA} \quad \text{(R4b)}$$

(5) HER (*H formation)

$$* + \mathrm{H_2O} + \mathrm{e^-} \rightarrow * \mathrm{H} + \mathrm{OH^-} \quad \text{(R5a)}$$

$$* + \mathrm{H_2O} + \mathrm{AA} + \mathrm{e^-} \rightarrow * \mathrm{H} + \mathrm{AA} + \mathrm{OH^-} \quad \text{(R5b)}$$

$$* + \mathrm{H_2O} + \mathrm{AA} + \mathrm{e^-} \rightarrow * \mathrm{H} + \mathrm{H_2O} + \mathrm{ASC^-} \quad \text{(R5c)}$$

(6) HER (H_2 production)

$$* \mathrm{H} + \mathrm{H_2O} + \mathrm{e^-} \rightarrow * + \mathrm{OH^-} + \mathrm{H_2}\ (\mathrm{g}) \quad \text{(R6a)}$$

$$* \mathrm{H} + \mathrm{H_2O} + \mathrm{AA} + \mathrm{e^-} \rightarrow * + \mathrm{OH^-} + \mathrm{AA} + \mathrm{H_2}\ (\mathrm{g}) \quad \text{(R6b)}$$

$$* \mathrm{H} + \mathrm{H_2O} + \mathrm{AA} + \mathrm{e^-} \rightarrow * + \mathrm{H_2O} + \mathrm{ASC^-} + \mathrm{H_2}\ (\mathrm{g}) \quad \text{(R6c)}$$

Functional groups in graphene quantum dots (GQDs) can affect CO_2RR.[3] However, in our work, the effect of GQDs on CO_2RR is not dominant for cAA-CuNW because most GQDs lost their functional groups by the reduction during AA introduction and the types of GQD functional groups are less controlled. Therefore, since the presence of GQDs is not expected to have a significant impact on the DFT results, we excluded GQDs from the simulation model.

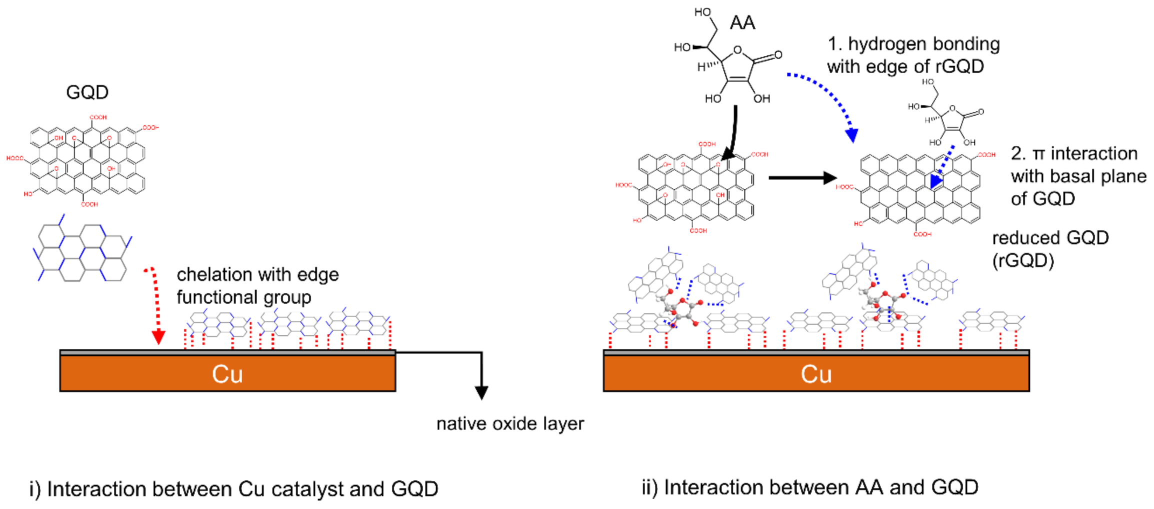

Supplementary Fig. 1 | Schematic illustration of the interaction between AA, GQDs, and Cu surface.

The functional groups of GQDs such as hydroxyl and carboxyl group can be interacted and chelated to the nanometer-scale native oxide layer on the Cu surface.[4, 5] In the reaction between GQD and AA, AA removes impure oxygenated functional group in GQD through nucleophilic substitution (S_N2) reaction and reduces GQD through thermal elimination.[6, 7] Since the reaction between AA and GQD proceeded for 1 h at 95°C, most intermediates formed during the S_N2 reaction were eliminated.

The reduced GQDs can be combined with AA via physisorption, such as π interaction at the basal plane or hydrogen bonding with edge functional groups. Molecules with endiol groups (e.g. catechol), carboxyl group, or aromatic rings can form 2-dimensional supramolecular systems with reduced graphene through π interaction or hydrogen bonding.[8-11]

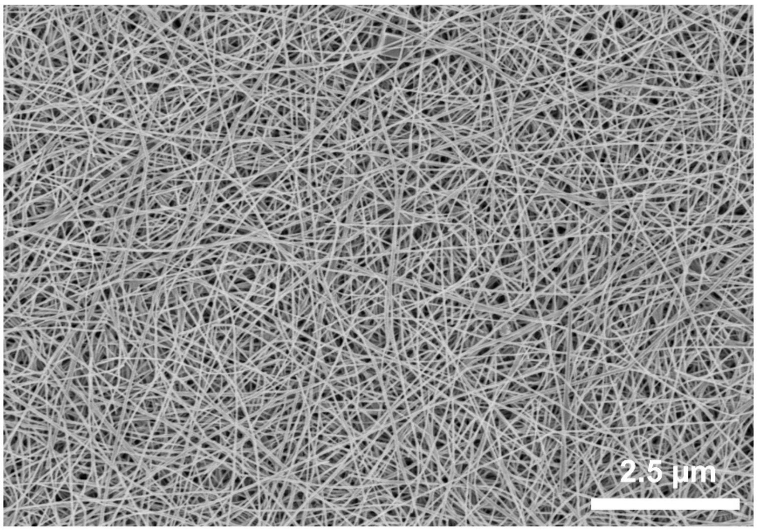

Supplementary Fig. 2 | SEM image of as-synthesized cAA-CuNW. SEM image shows the surface structure and uniform distribution of CuNWs with AA and GQDs.

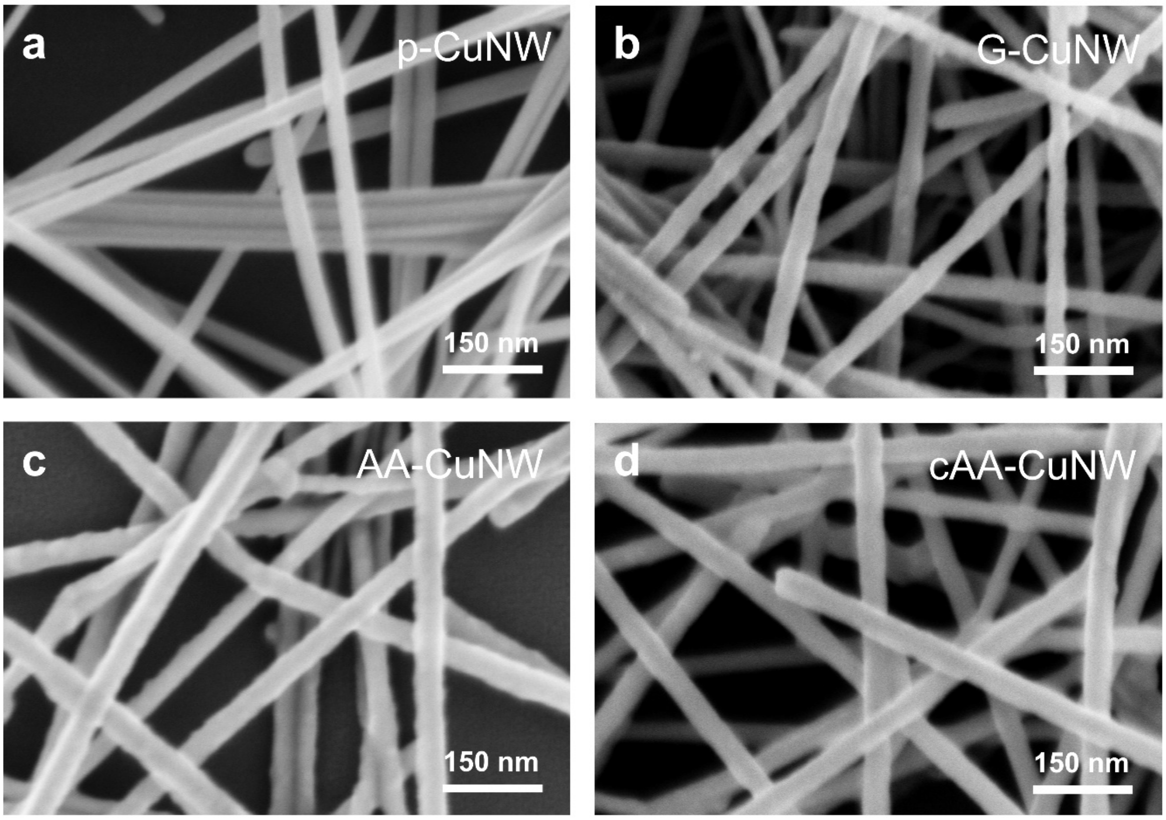

Supplementary Fig. 3 | SEM images of as-synthesized CuNWs according to the degree of surface hybridization. SEM images of (**a**) p-CuNW, (**b**) G-CuNW, (**c**) AA-CuNW, and (**d**) cAA-CuNW. All CuNWs were prepared by a sonication-mediated wrapping method. There was no significant deformation in their one-dimensional (1D) morphology.

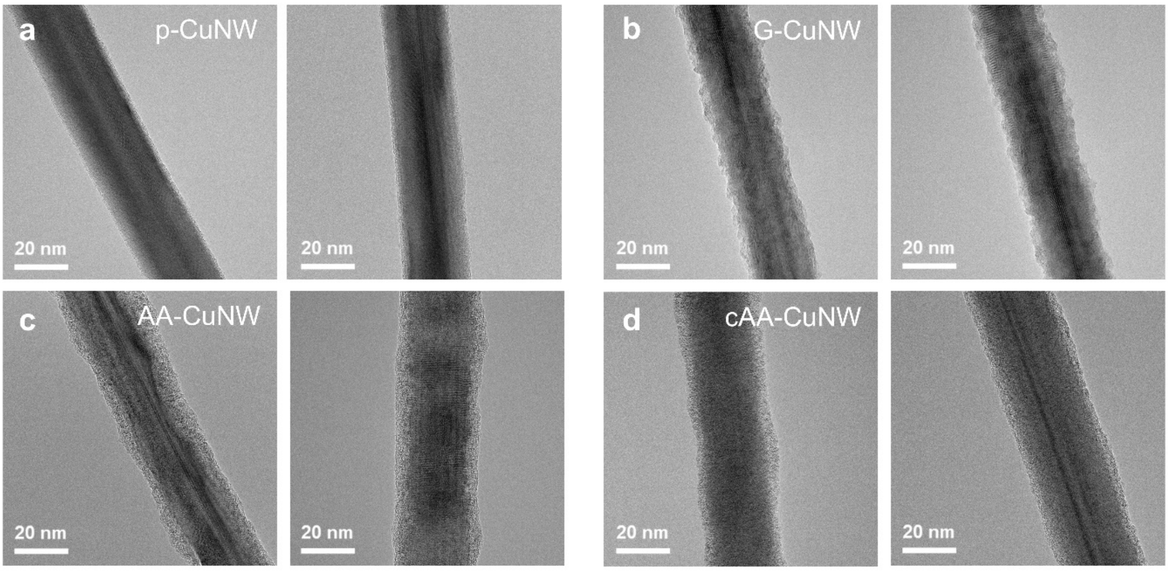

Supplementary Fig. 4 | TEM images of as-synthesized CuNW according to the degree of surface hybridization. The surface nanostructure of each CuNW was investigated as TEM images of (**a**) p-CuNW, (**b**) G-CuNW, (**c**) AA-CuNW, and (**d**) cAA-CuNW.

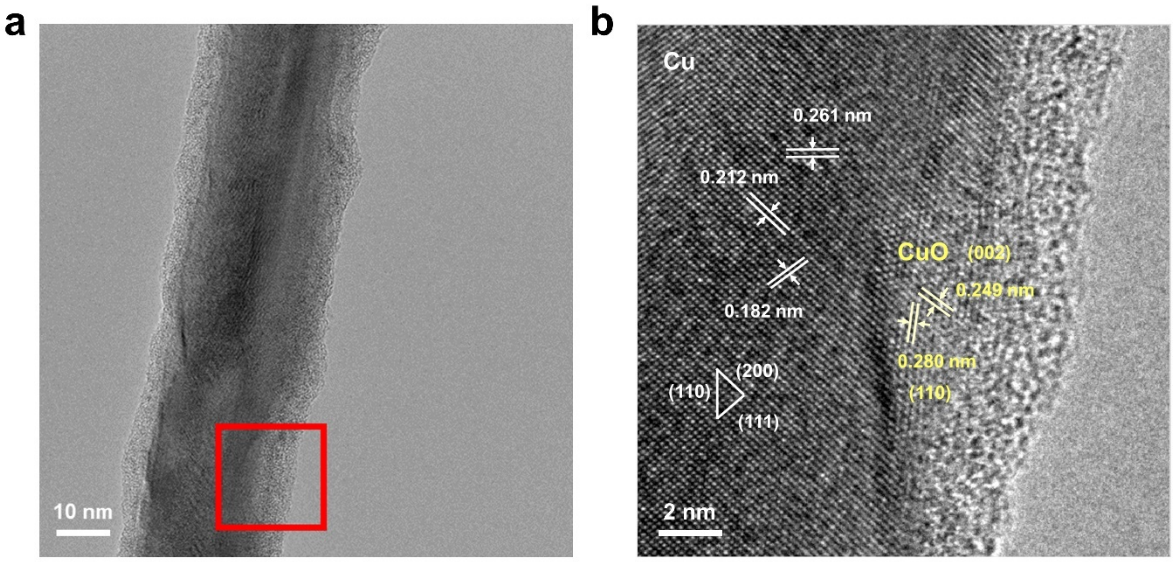

Supplementary Fig. 5 | Surface Cu oxidation during the synthesis of AA-CuNW. a TEM image of the oxidized AA-CuNW. **b** Magnified TEM image at the surface area of AA-CuNW.

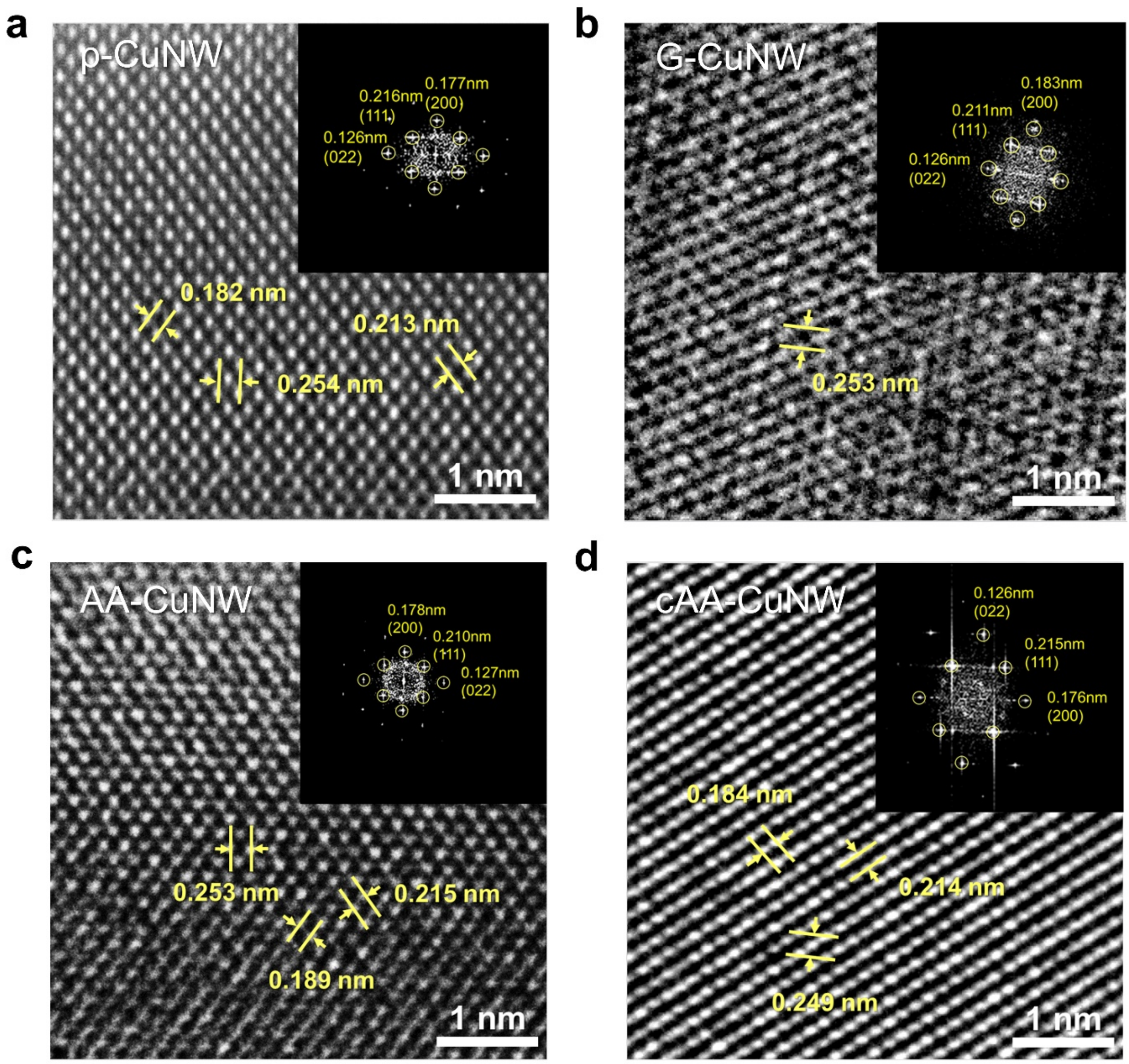

Supplementary Fig. 6 | High-resolution (HR) TEM images of CuNW to investigate the crystalline phase and atomic arrangement. Crystal structures of the core Cu in (**a**) p-CuNW, (**b**) G-CuNW, (**c**) AA-CuNW, and (**d**) cAA-CuNW. Inset: Fast Fourier transform (FFT) image of the corresponding pure Cu phase.

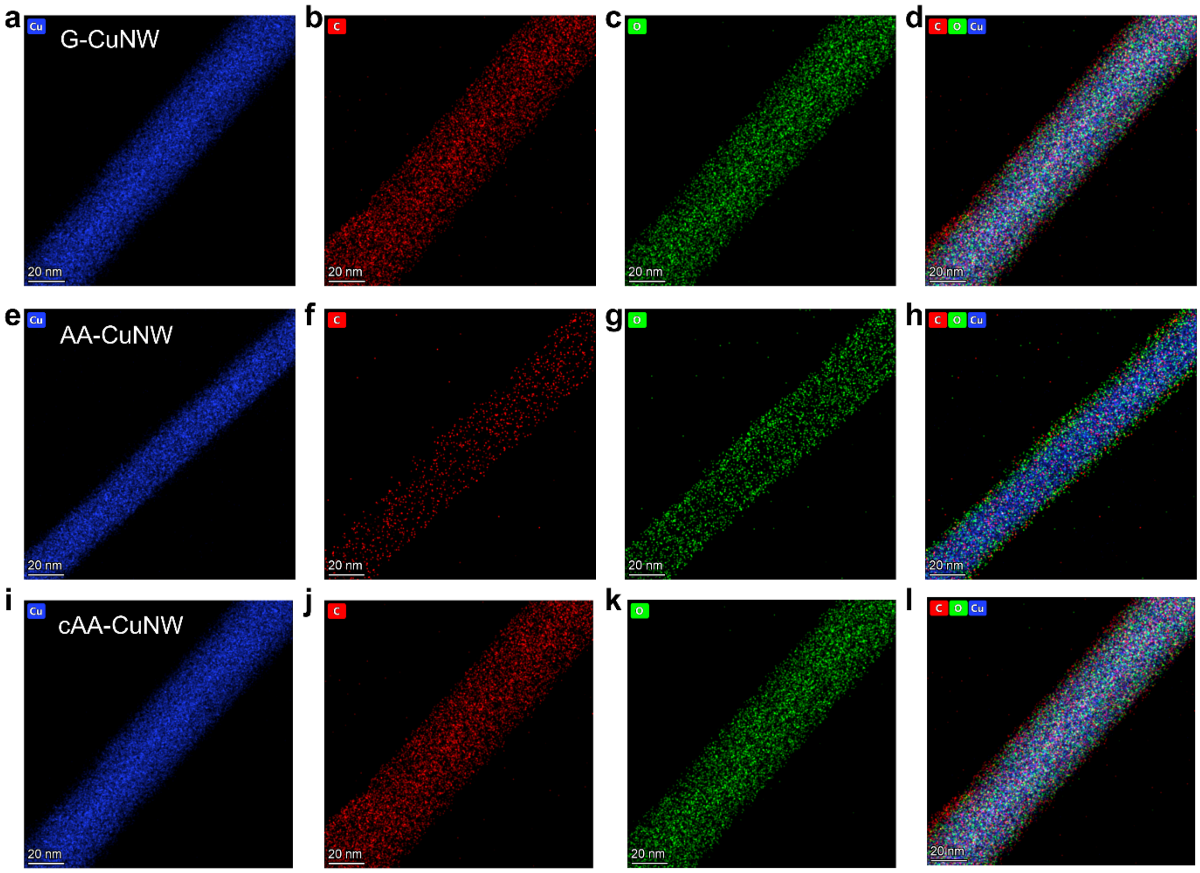

Supplementary Fig. 7 | Cu, C, O elemental distributions of CuNWs. TEM EDS mapping of Cu, C, O, and the combination of Cu-C-O for (**a–d**) G-CuNW, (**e–h**) AA-CuNW, and (**i–l**) cAA-CuNW, respectively.

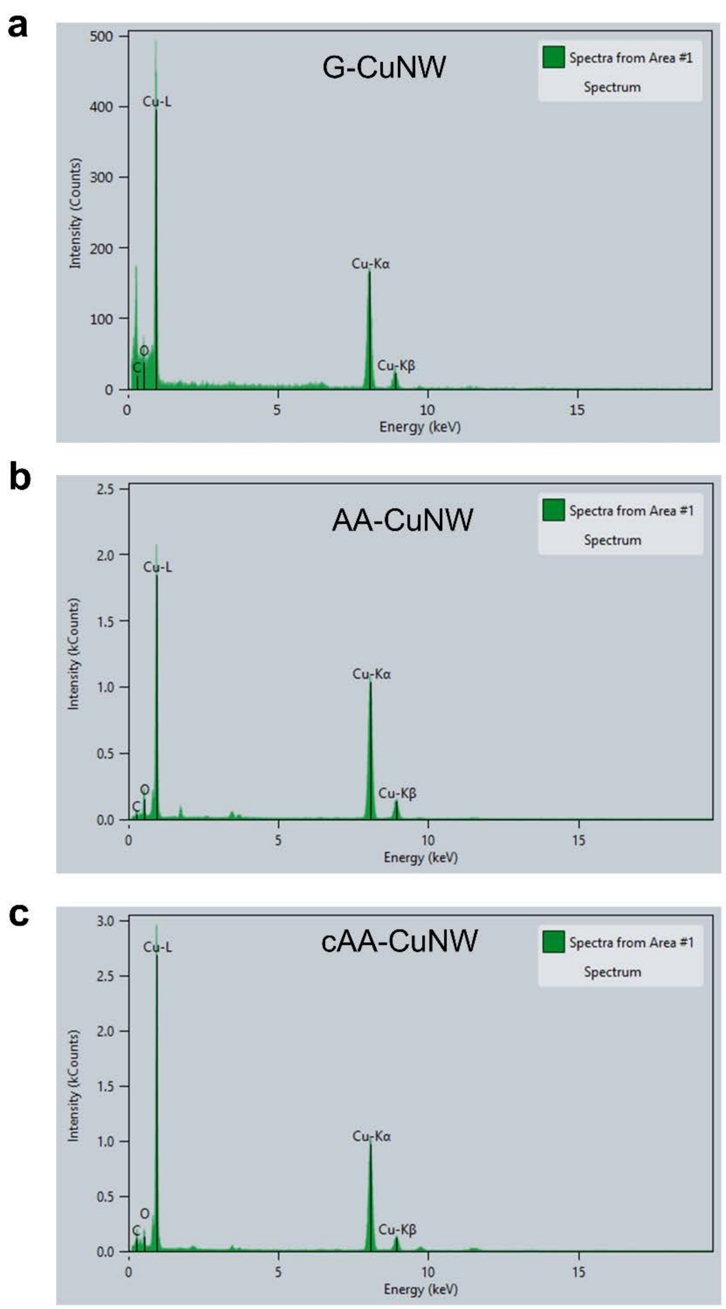

Supplementary Fig. 8 | TEM EDS spectrum of CuNWs for atomic faction analysis. Atomic elements of Cu, C, and O were detected in (**a**) G-CuNW, (**b**) AA-CuNW, and (**c**) cAA-CuNW.

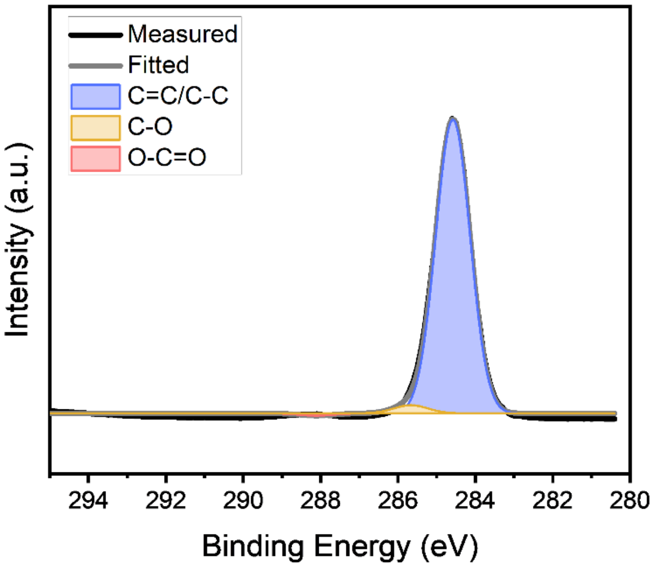

Supplementary Fig. 9 | C 1*s* XPS spectra of GQDs separated from cAA-CuNW. To investigate the oxidation states and chemical bonding of GQD in cAA-CuNW, GQDs were separated from cAA-CuNW via repeated ultrasonication and centrifugation.

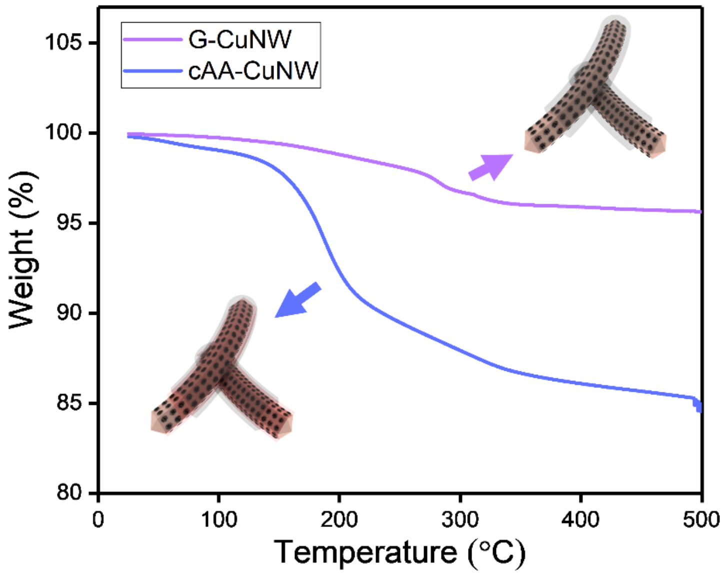

Supplementary Fig. 10 | TGA analysis of G-CuNW and cAA-CuNW to verify the presence of AA in cAA-CuNW. The large weight loss of cAA-CuNW near 190°C is due to the decomposition of AA.

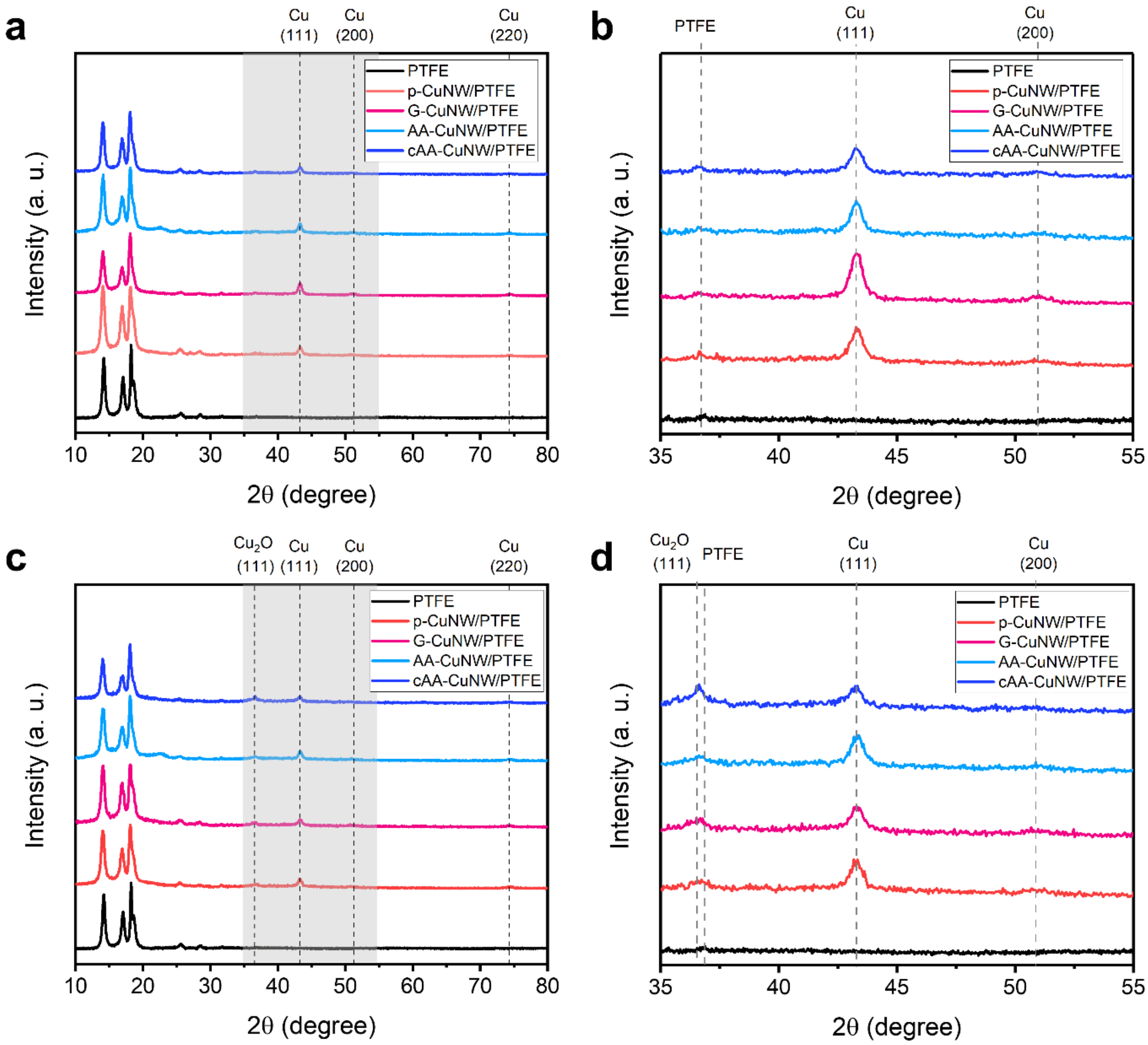

Supplementary Fig. 11 | Crystal structure analysis of CuNWs after CO_2RR. a XRD patterns of p-CuNW, G-CuNW, AA-CuNW, and cAA-CuNW on PTFE substrate before CO_2RR. **b** Magnified XRD patterns of each sample before CO_2RR. **c** XRD patterns of p-CuNW, G-CuNW, AA-CuNW, and cAA-CuNW on PTFE substrate after CO_2RR. **d** Magnified XRD patterns of each sample after CO_2RR.

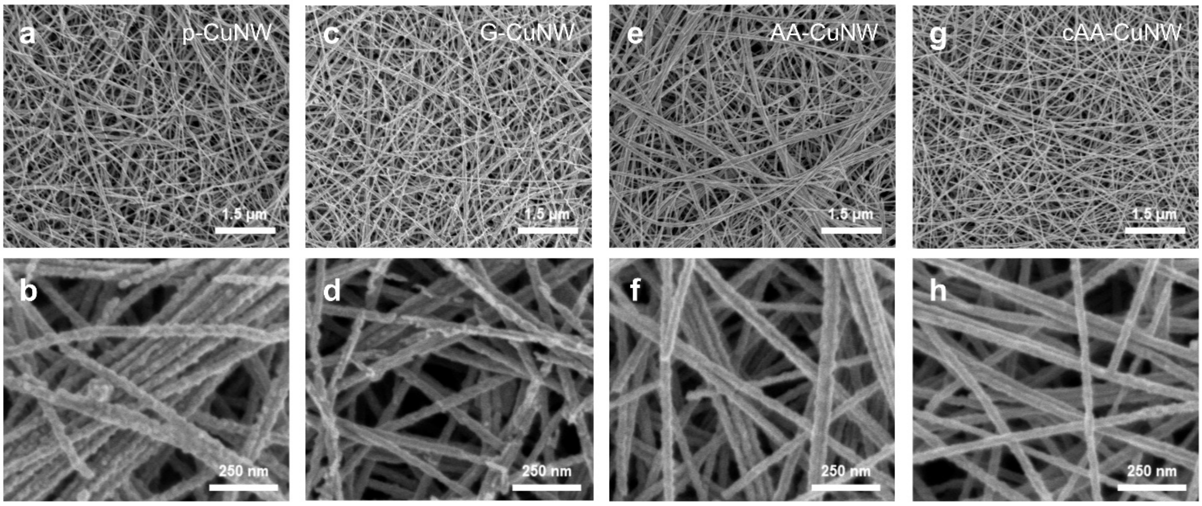

Supplementary Fig. 12 │ Investigation on microstructures of CuNWs after CO_2RR. SEM (top) and HR-SEM (bottom) images of (**a**, **b**) p-CuNW, (**c**, **d**) G-CuNW, (**e**, **f**) AA-CuNW, and (**g**, **h**) cAA-CuNW after CO_2RR.

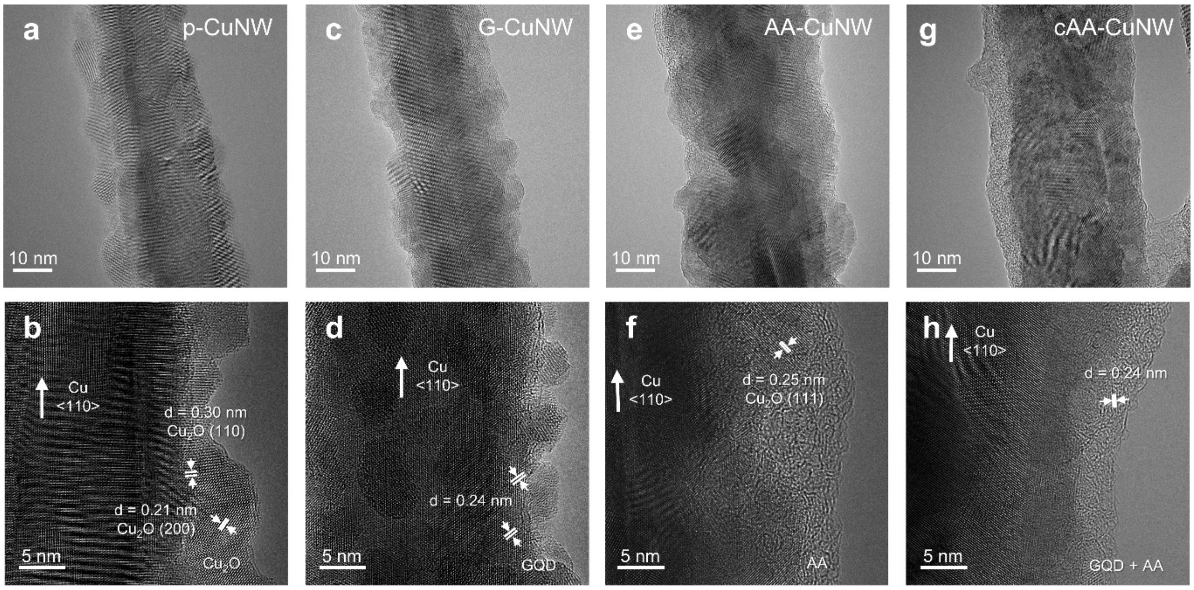

Supplementary Fig. 13 | Investigation on surface structures of CuNWs after CO_2RR. TEM (top) and HR-TEM (bottom) images of (**a**, **b**) p-CuNW, (**c**, **d**) G-CuNW, (**e**, **f**) AA-CuNW, and (**g**, **h**) cAA-CuNW after CO_2RR.

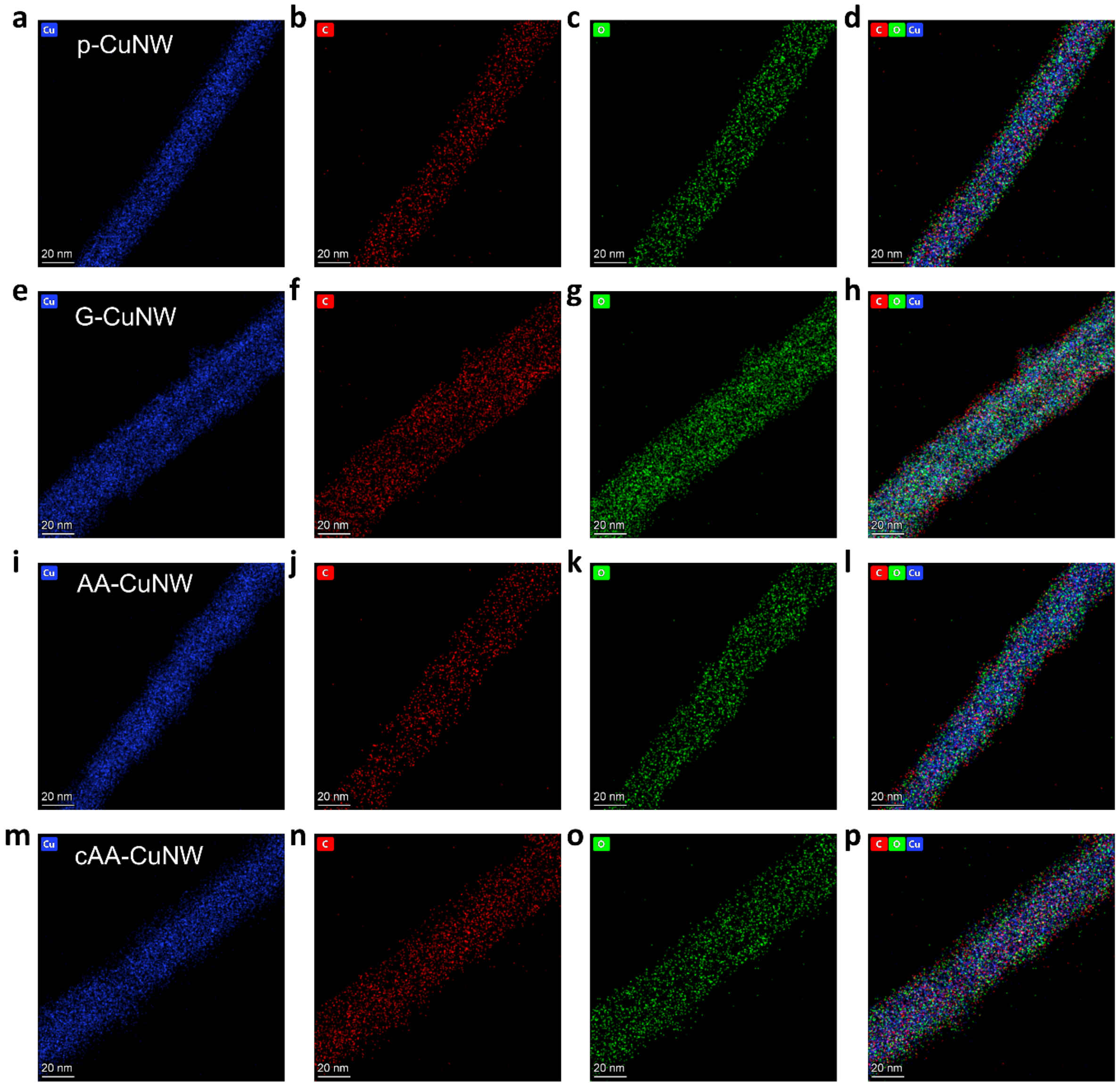

Supplementary Fig. 14 | Investigation on elemental distribution of CuNWs after CO_2RR. TEM EDS mapping of Cu, C, O, and the combination of Cu-C-O for (**a–d**) p-CuNW, (**e–h**) G-CuNW, (**i–l**) AA-CuNW, and (**m–p**) cAA-CuNW after CO_2RR, respectively.

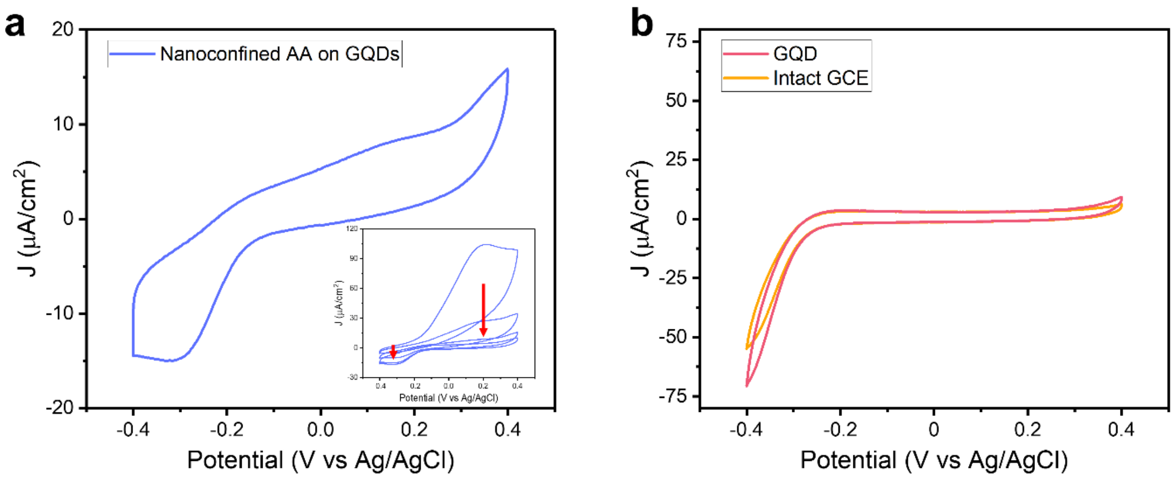

Supplementary Fig. 15 | Redox behavior of nanoconfined AA on GQDs without gas supply. **a** Cyclic voltammetry (CV) of a GCE coated with nanoconfined AA on GQDs in a 1 M KOH electrolyte. Inset: 2nd cycle to 5th cycle CV of the GCE coated with nanoconfined AA on GQDs. As the cycle progressed, the integration of the oxidation peak (0.18 V vs Ag/AgCl) gradually decreased, while that of the reduction peak (–0.30 V vs Ag/AgCl) increased. **b** CVs from the intact GCE and GCE coated with GQDs. All CVs were taken from the 3rd cycle.

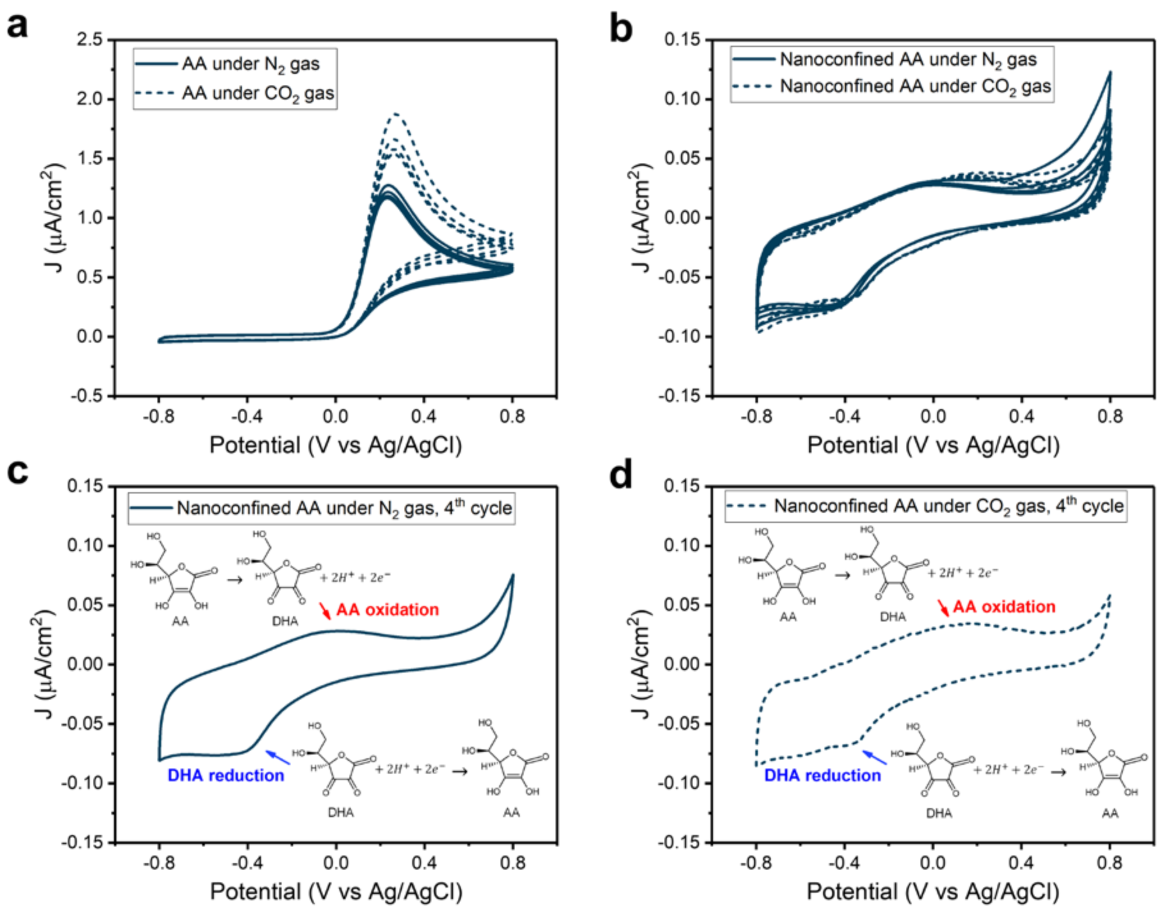

Supplementary Fig. 16 | Redox behavior of AA and nanoconfined AA on GQDs under N_2 and CO_2 gas. a CV plot of GCE with AA dissolved in 0.1 M $KHCO_3$ electrolyte. **b** CV plot of GCE coated with nanoconfined AA on GQDs in 0.1 M $KHCO_3$ electrolyte. 4th cycle of the CV plot of GCE coated with nanoconfined AA on GQDs under (**c**) N_2 and (**d**) CO_2 gas.

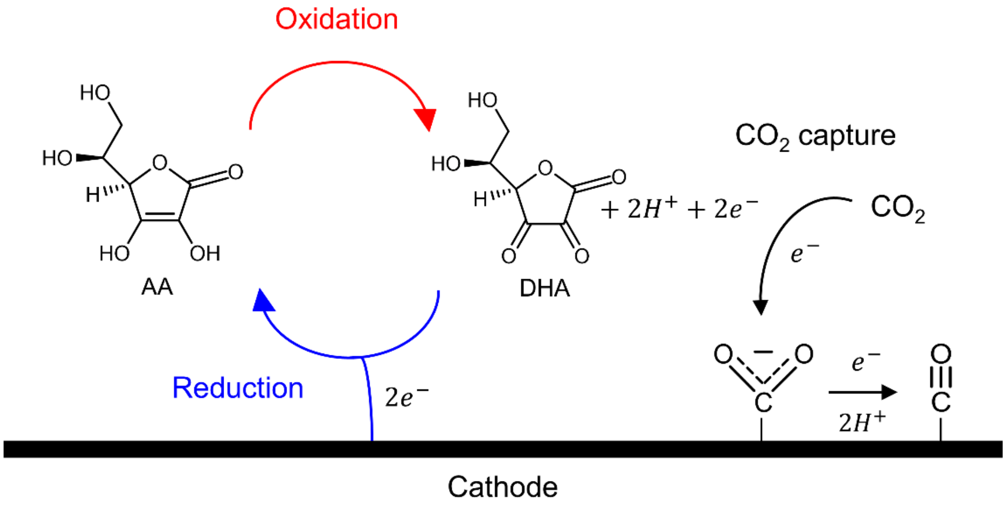

Supplementary Fig. 17 | Schematic illustration of our strategy for CO_2 capture in atmosphere. The redox behavior of nanoconfined AA on GQDs contributes to CO_2 capture by promoting CO_2-to-*CO conversion during CO_2RR.

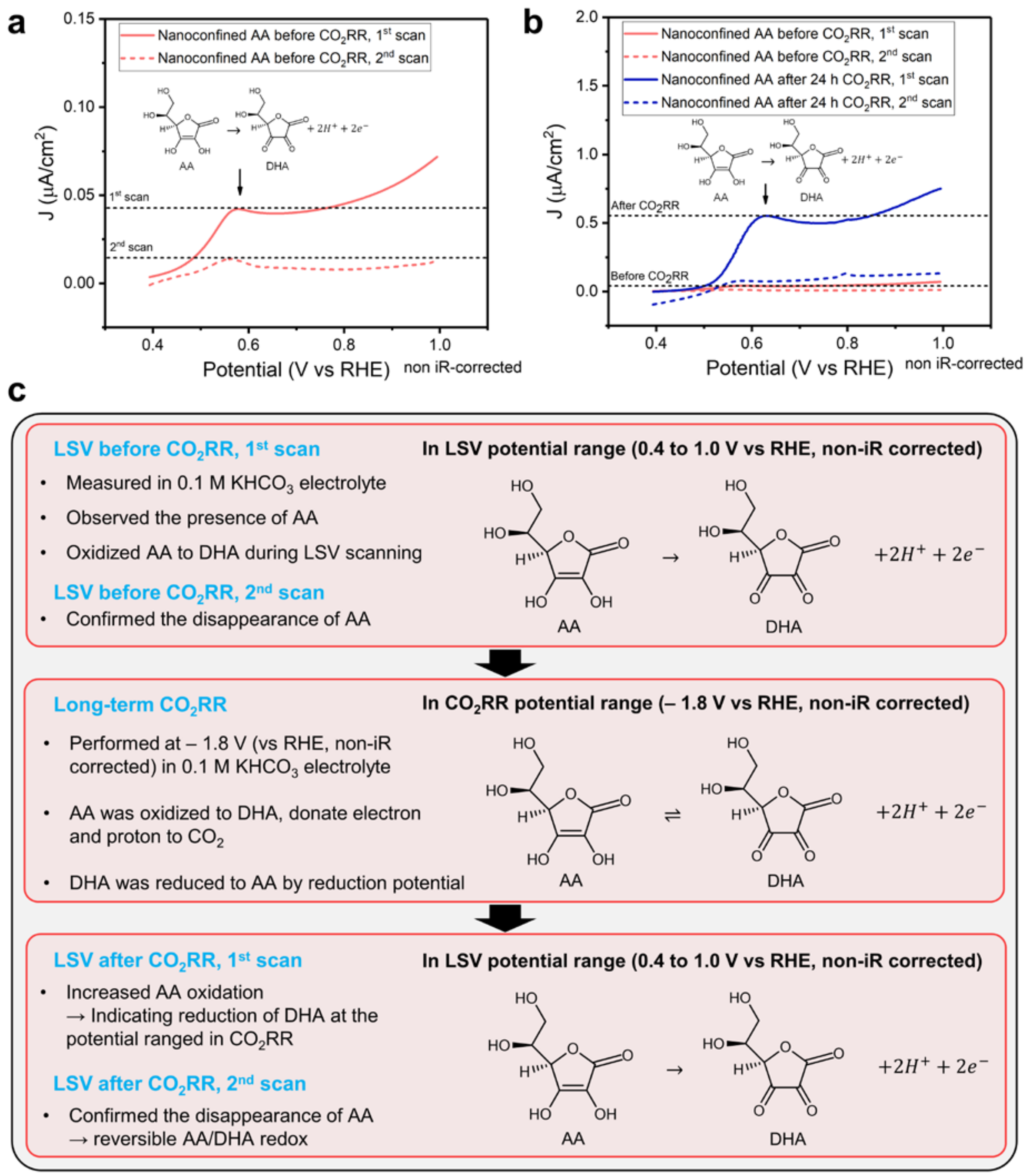

Supplementary Fig. 18 | Linear sweep voltammetry (LSV) of nanoconfined AA on GQDs before and after CO_2RR. LSV of nanoconfined AA on GQDs (**a**) before and (**b**) after CO_2RR in 0.1 M $KHCO_3$ electrolyte. **c** Flow chart to investigate the redox behavior of AA/DHA in the potential range of LSV and CO_2RR. Nanoconfined AA on GQDs was prepared on GCE and CO_2RR was conducted at a constant potential of –1.8 V (vs RHE, non-iR corrected) in 0.1 M $KHCO_3$ electrolyte.

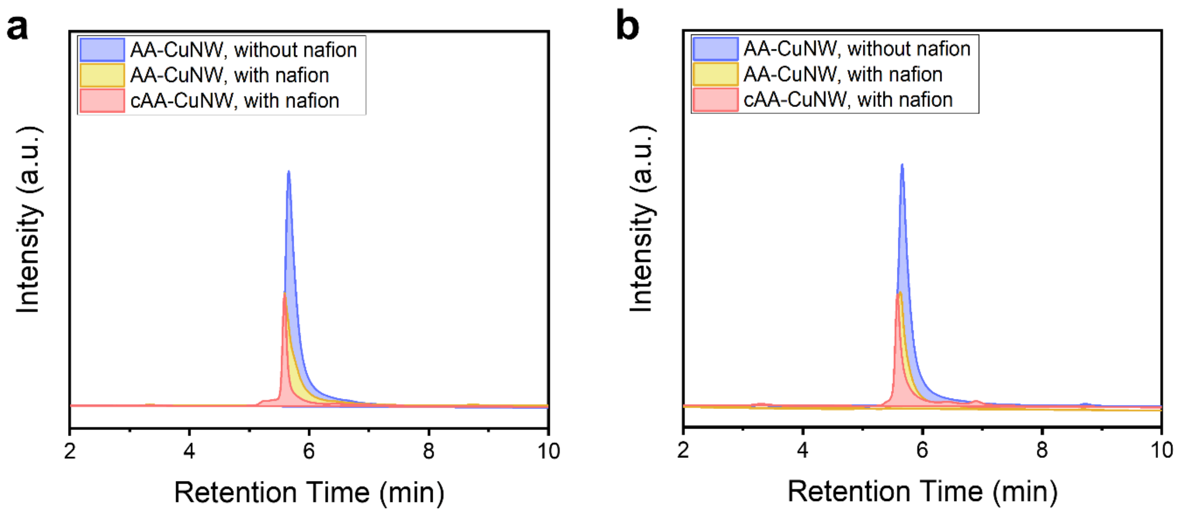

Supplementary Fig. 19 | HPLC analysis of the extracted solution from AA- and cAA-CuNW to verify the immobilization effect of Nafion on AA. Chromatographs of the extracted solution with extraction times of (**a**) 30 and (**b**) 60 min. The integration of the peak is much larger for CuNW without Nafion.

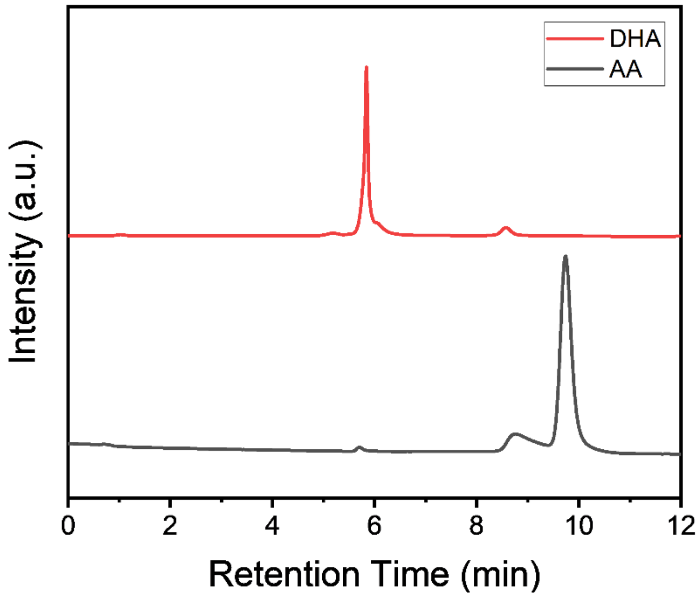

Supplementary Fig. 20 | HPLC analysis of standard solutions of AA and dehydroascorbic acid (DHA). The chromatograph shows that the peak with a retention time of ~5.8 min corresponds to DHA under the same HPLC conditions.

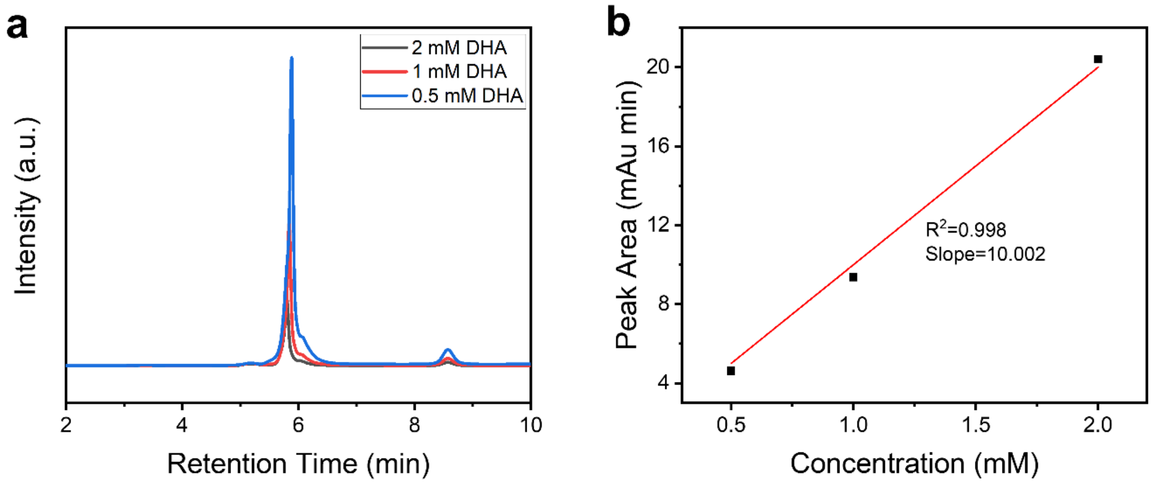

Supplementary Fig. 21 | HPLC calibration data of DHA. a Chromatograph of DHA solution with concentrations of 0.5, 1, and 2 mM. **b** Calibration curve for DHA. The correlation coefficient (R^2) for DHA was 0.998.

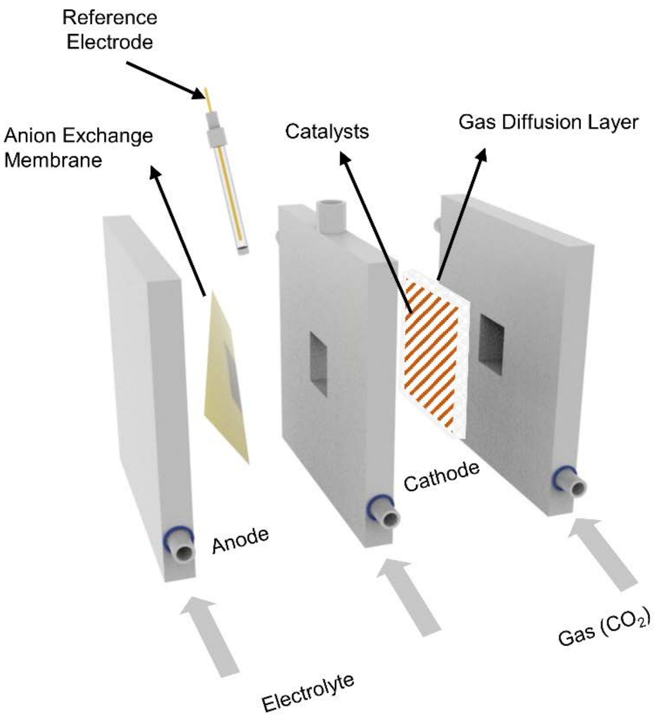

Supplementary Fig. 22 | Schematic of flow cell components including electrodes and membrane for electrochemical CO_2RR. Electrolytes for the cathode and anode are circulated independently, and CO_2 gas is supplied directly to the gas diffusion electrode.

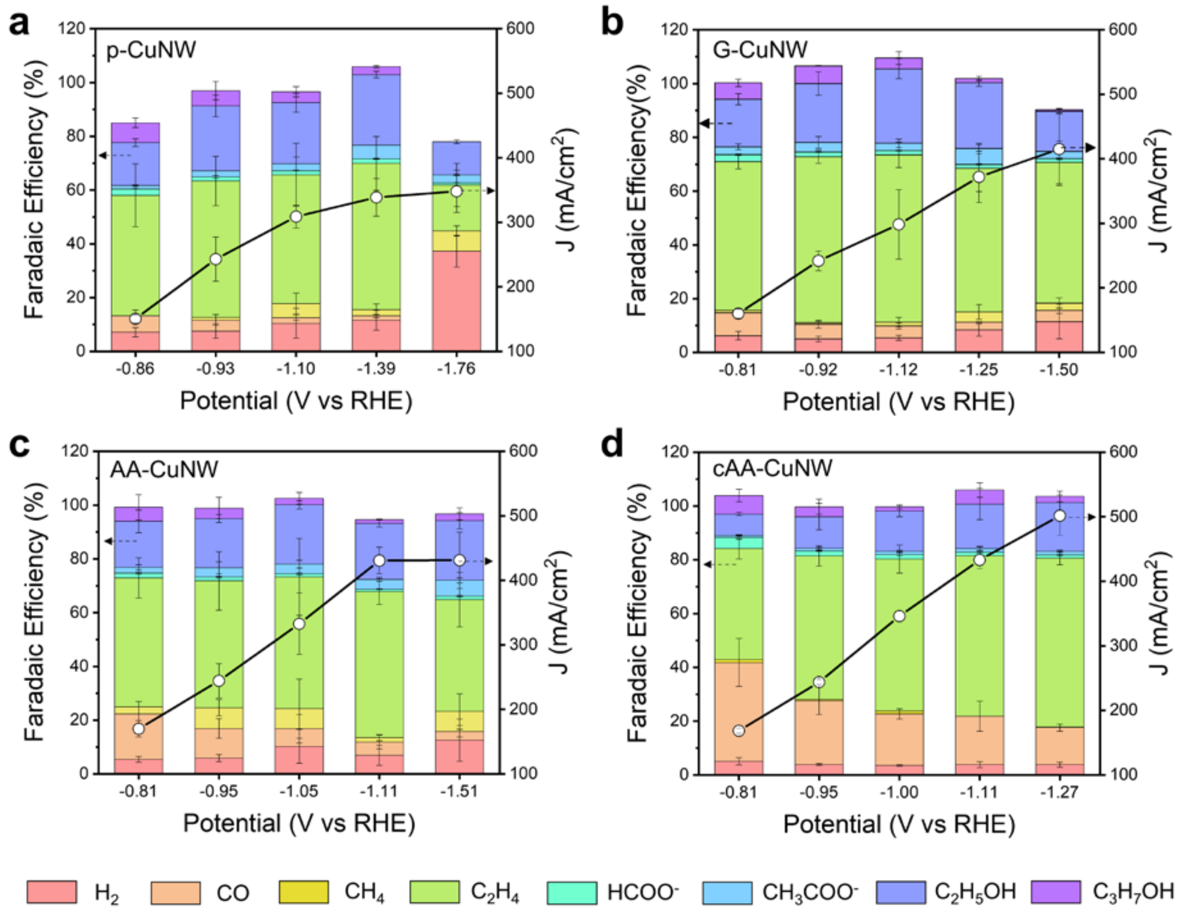

Supplementary Fig. 23 | Electrochemical CO_2RR performance of CuNWs. Faradaic efficiencies (FEs) and current densities for (**a**) p-CuNW, (**b**) G-CuNW, (**c**) AA-CuNW, and (**d**) cAA-CuNW. All tests were performed in a flow cell electrolyzer with 1 M KOH electrolyte. All the error bars represent standard deviation based on three independent samples.

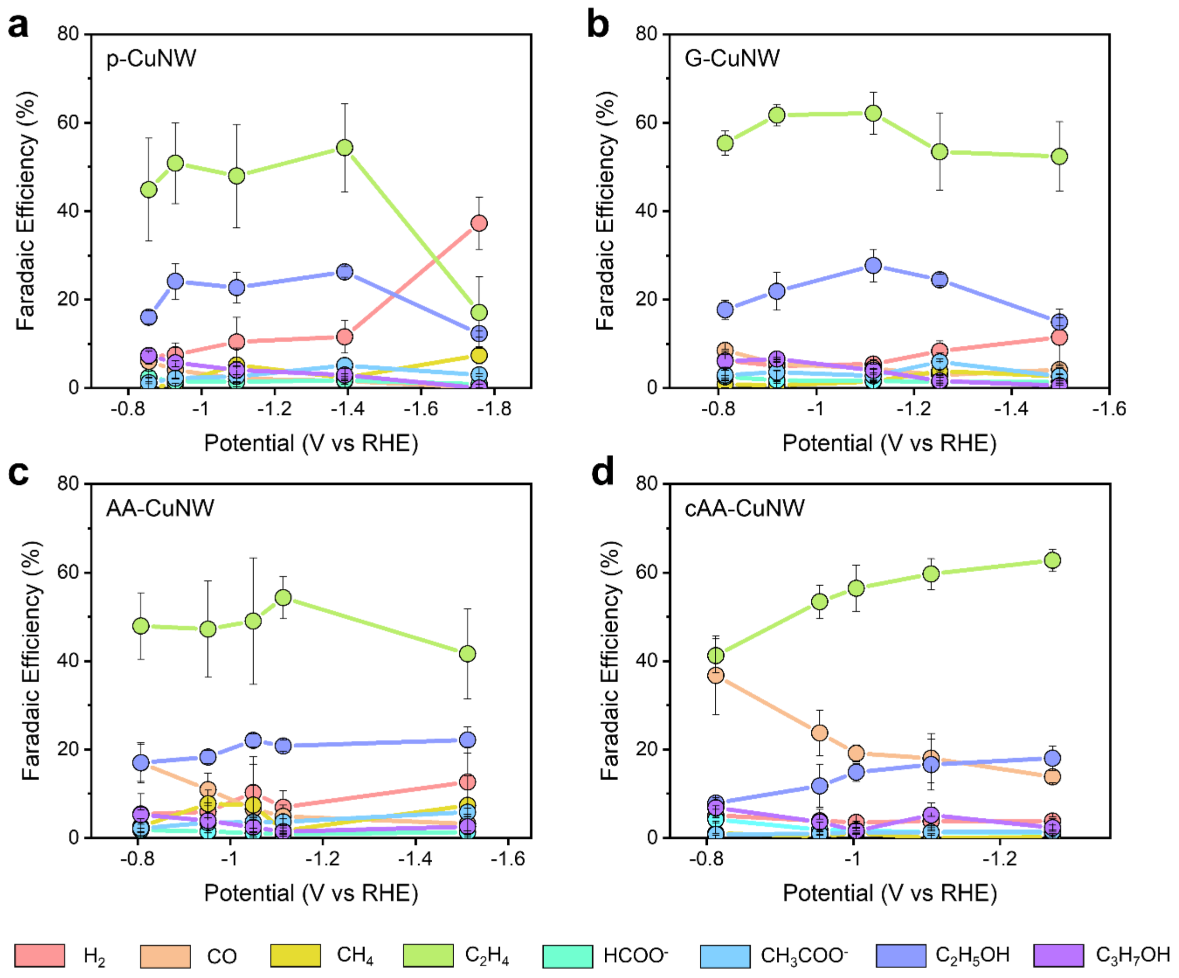

Supplementary Fig. 24 | Comparisons of the product selectivities. FEs of CO_2RR products for (**a**) p-CuNW, (**b**) G-CuNW, (**c**) AA-CuNW, and (**d**) cAA-CuNW. All the error bars represent standard deviation based on three independent samples.

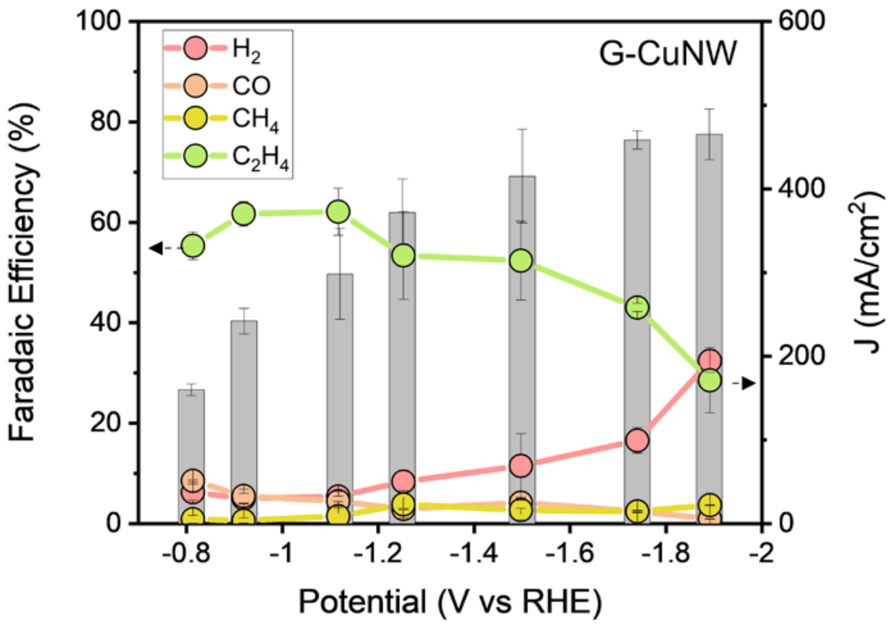

Supplementary Fig. 25 | Investigation of CO_2RR of G-CuNW at extended potential range. Gaseous product FEs and total current densities for G-CuNW up to –1.89 V (vs RHE). All the error bars represent standard deviation based on three independent samples.

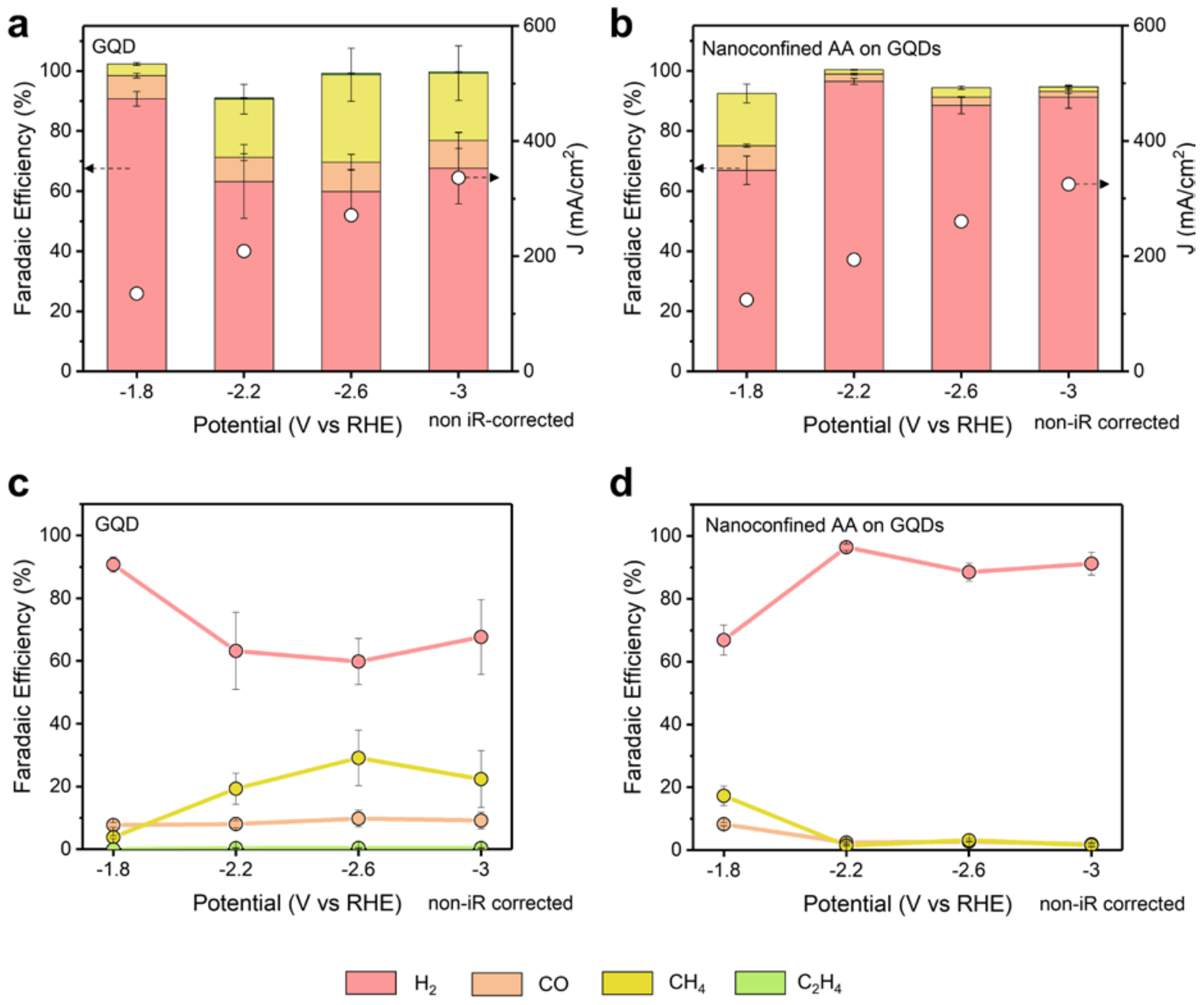

Supplementary Fig. 26 | Gaseous product selectivity of hybridization materials without CuNW. FEs of the gaseous products of (**a, c**) pristine GQD and (**b, d**) nanoconfined AA on GQDs. The electrodes were fabricated by spray-coating each material on porous carbon paper-based substrates. All the error bars represent standard deviation based on three independent measurements.

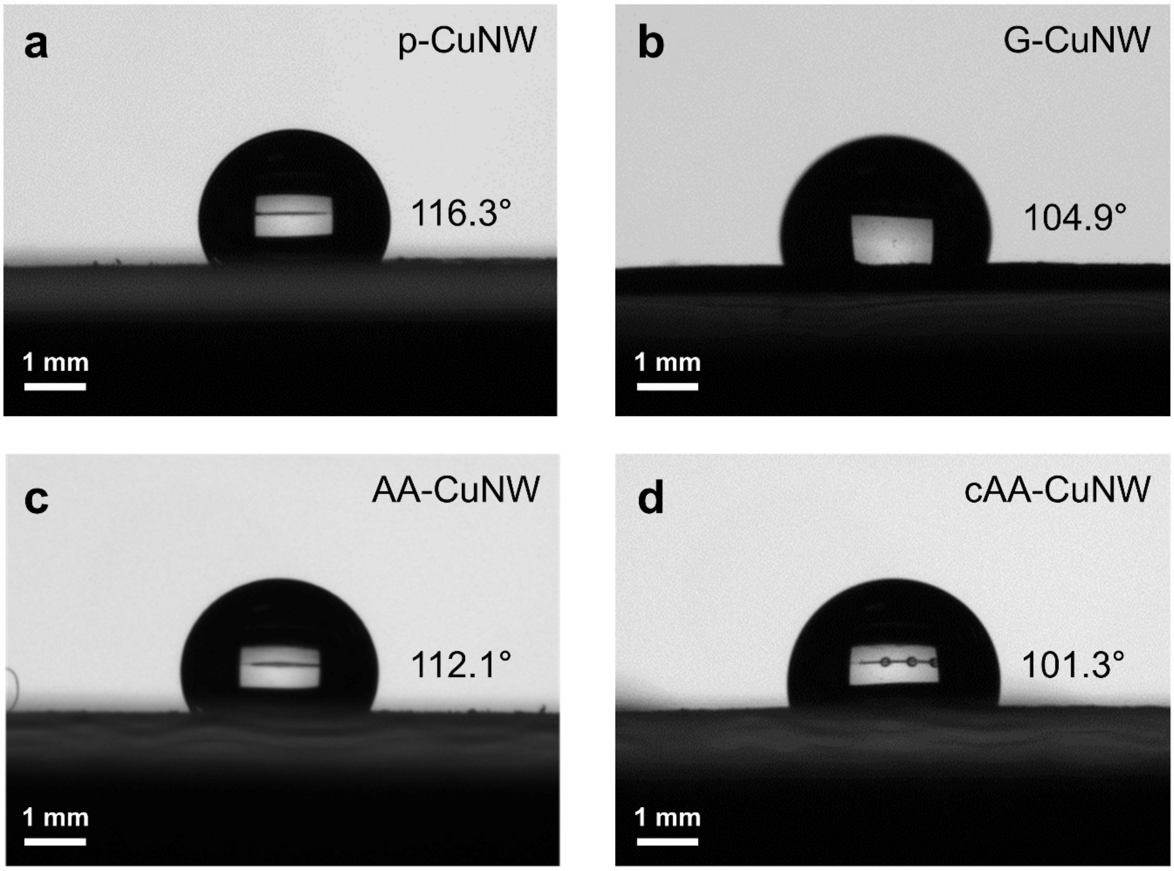

Supplementary Fig. 27 | Wetting properties of the CuNWs. Water contact angle of (**a**) p-CuNW, (**b**) G-CuNW, (**c**) AA-CuNW, and (**d**) cAA-CuNW on PTFE substrates.

Hydrophobicity is an important property of molecular additives in enhancing CO_2 mass transport. Improving the hydrophobicity of the catalyst surface can increase *CO coverage by participating in kinetic control between *CO and *H.[12] However, GQD is hydrophilic due to large amount of oxygen containing functional groups on edge site.[13] The water contact angles of G-CuNW and cAA-CuNW were slightly lower than others due to hydrophilic properties of GQDs. Therefore, the hydrophilic properties of GQDs are not beneficial for high current density CO_2RR.

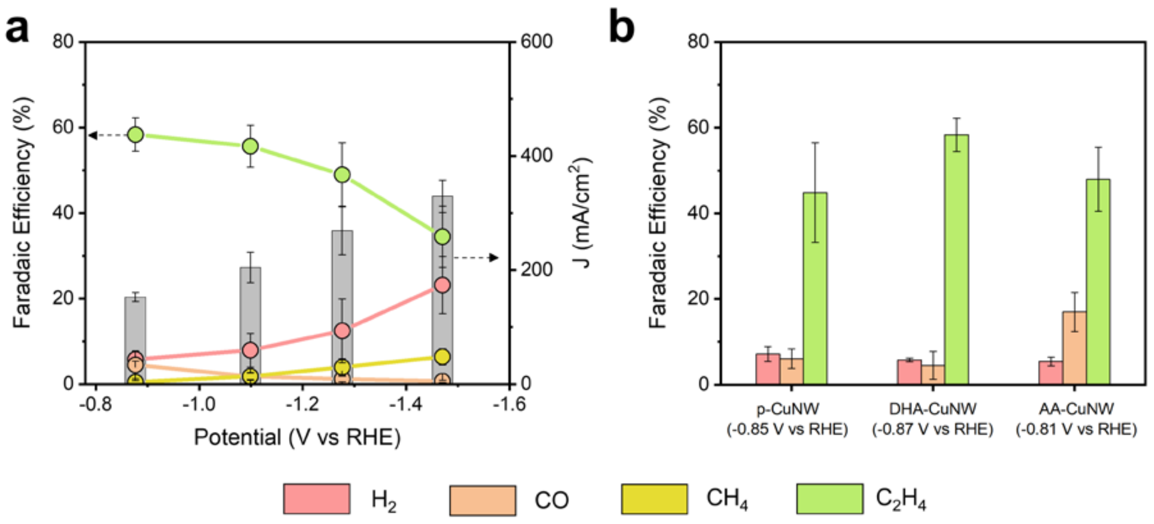

Supplementary Fig. 28 | Effect of DHA on electrochemical CO_2RR performance of CuNW. a Gaseous product FEs and total current densities of DHA-CuNW up to –1.47 V (vs RHE). **b** Comparison of the gaseous product selectivity of p-CuNW, DHA-CuNW, and AA-CuNW. All the error bars represent standard deviation based on three independent samples.

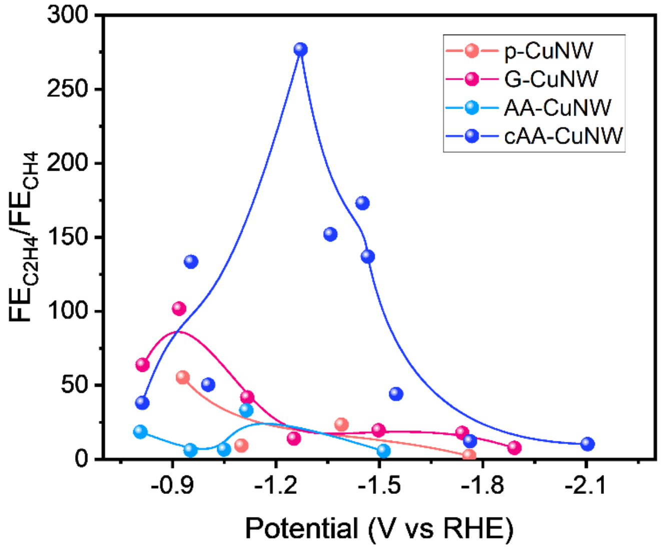

Supplementary Fig. 29 | Comparison of the C_2H_4 and CH_4 selectivity of CuNWs. The ratio of C_2H_4 FE to CH_4 FE (C_2H_4 FE/CH_4 FE) in p-CuNW, G-CuNW, AA-CuNW, and cAA-CuNW according to applied potentials.

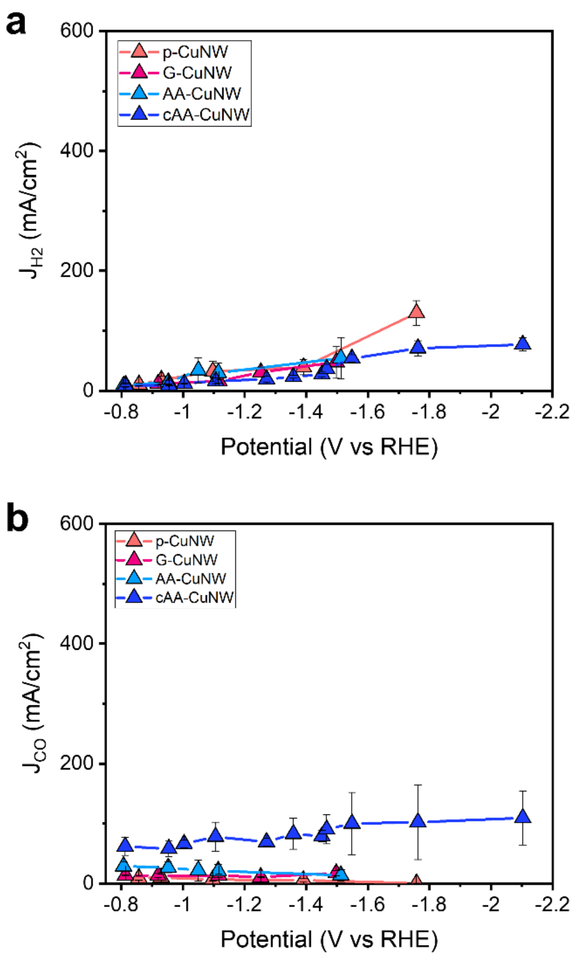

Supplementary Fig. 30 | Comparisons of the H_2 and CO productivity of CuNWs in CO_2 electrolysis. Partial current densities versus potentials of p-CuNW, G-CuNW, AA-CuNW, and cAA-CuNW were compared in terms of (**a**) H_2 and (**b**) CO. All the error bars represent standard deviation based on three independent samples.

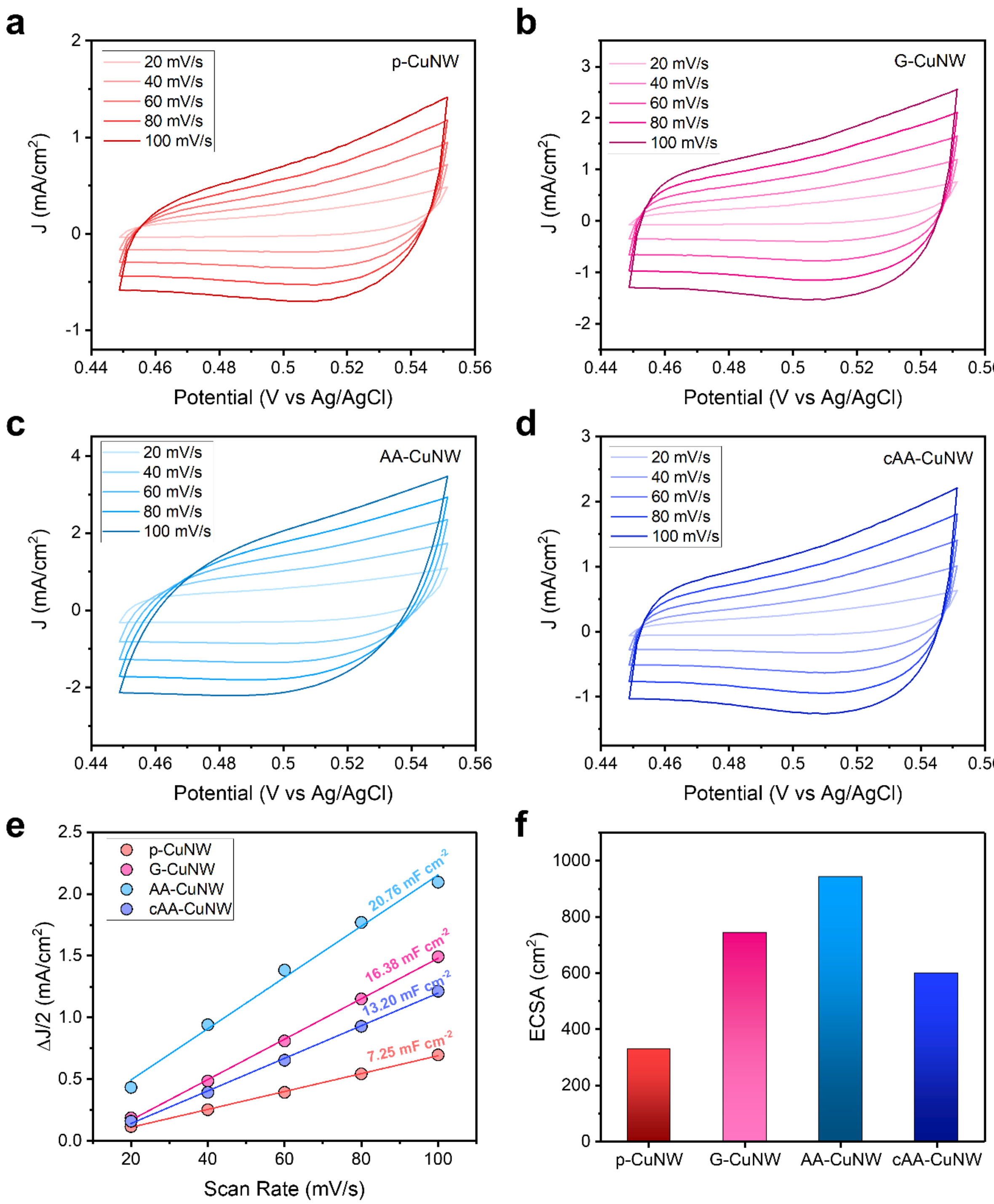

Supplementary Fig. 31 | Electrochemically active surface area (ECSA) measurement for CuNWs. CV plot of (**a**) p-CuNW, (**b**) G-CuNW, (**c**) AA-CuNW, and (**d**) cAA-CuNW in 1 M KOH electrolyte. **e** electrochemical double-layer capacitance (C_{dl}) and (**f**) the calculated ECSA for CuNWs. The ECSA was characterized by calculation of C_{dl} over specific capacitance (C_{s}), and C_{dl} was determined from the equation: $C_{\mathrm{dl}} = \Delta j(j_{\mathrm{a}} - j_{\mathrm{c}})/2v$, where j_{a} and j_{c} are anodic and cathodic current densities and v is the scan rate. C_{s} in 1 M KOH electrolyte was assumed as 0.022 mF/cm^2.

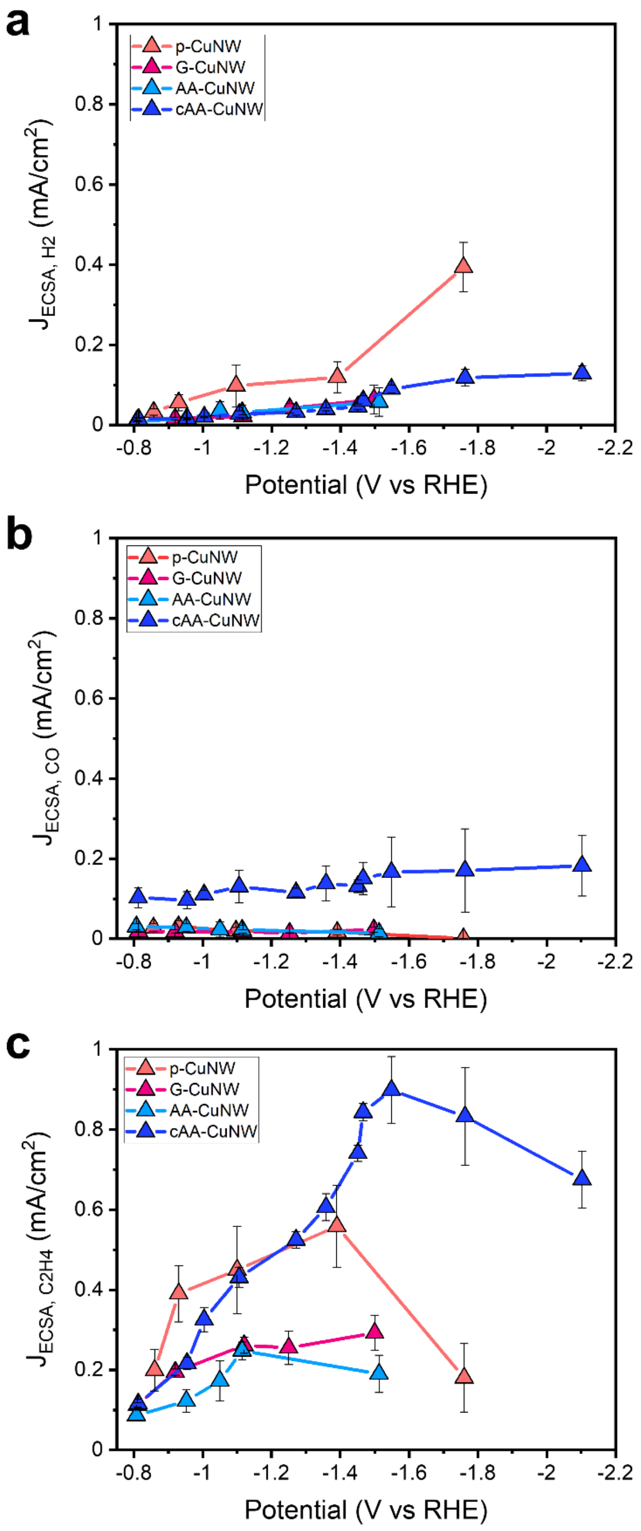

Supplementary Fig. 32 | Comparison of ECSA-normalized CO_2RR productivities between CuNWs. Partial current densities versus potentials of p-CuNW, G-CuNW, AA-CuNW, and cAA-CuNW were compared in terms of (**a**) H_2, (**b**) CO, and (**c**) C_2H_4. All the error bars represent standard deviation based on three independent samples.

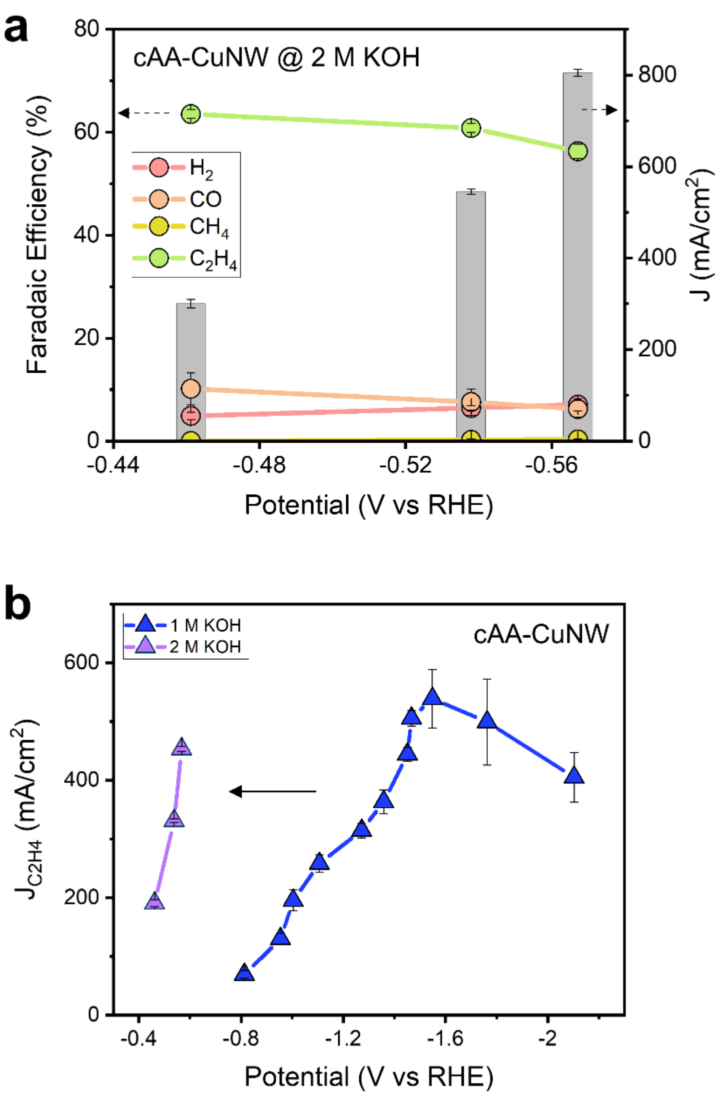

Supplementary Fig. 33 | Effect of KOH concentration on shifting the potential of high-rate C_2H_4 production of cAA-CuNW. a Gaseous product FEs and total current densities for cAA-CuNW with 2 M KOH electrolyte. **b** Comparison of J_{C2H4} versus potentials of cAA-CuNW according to the KOH concentration. All the error bars represent standard deviatio n based on three independent samples.

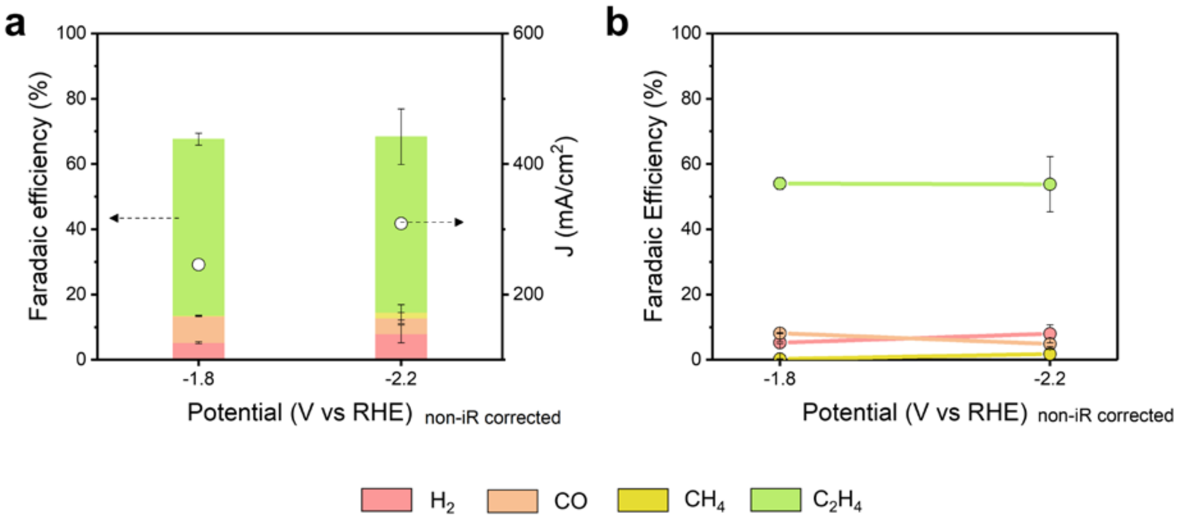

Supplementary Fig. 34 | Gaseous product selectivity of CuNW modified with AA and GQD without a preceding reaction. a Gaseous product FEs and total current densities of CuNW modified with AA and GQD without a preceding reaction. **b** Line plot of gaseous product FEs of CuNW modified with AA and GQD without a preceding reaction. The modified CuNW was prepared by wrapping with AA and GQD mixed solution through a sonication-mediated method without chemical reaction to compare with the CO_2RR performance of cAA-CuNW. All the error bars represent standard deviation based on three independent measurements.

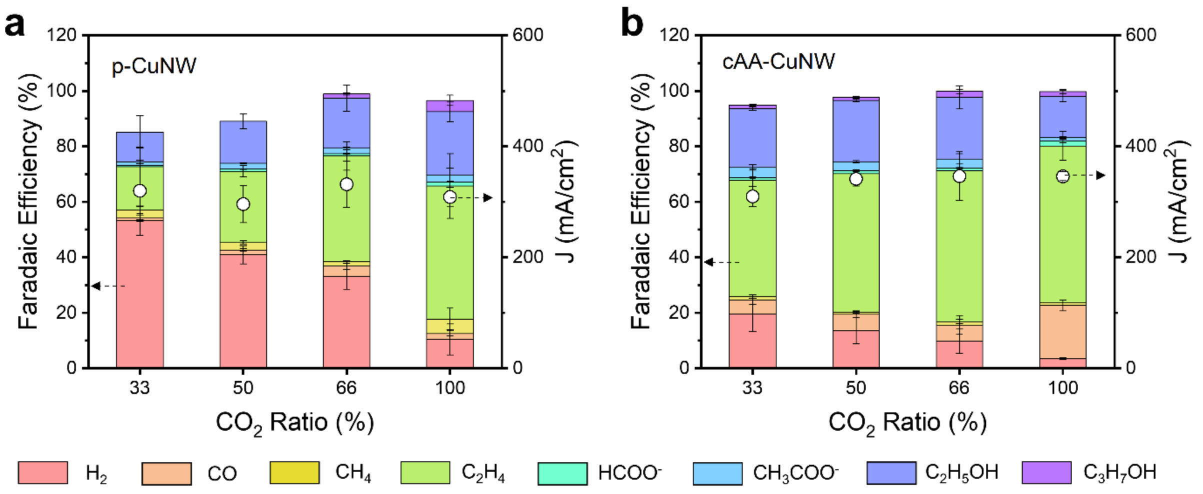

Supplementary Fig. 35 | Investigation of CO_2RR at low CO_2 concentration. Gaseous product FEs and total current densities of (**a**) p-CuNW and (**b**) cAA-CuNW in the CO_2RR according to CO_2 ratios in CO_2+Ar mixed gas. All the error bars represent standard deviation based on three independent samples.

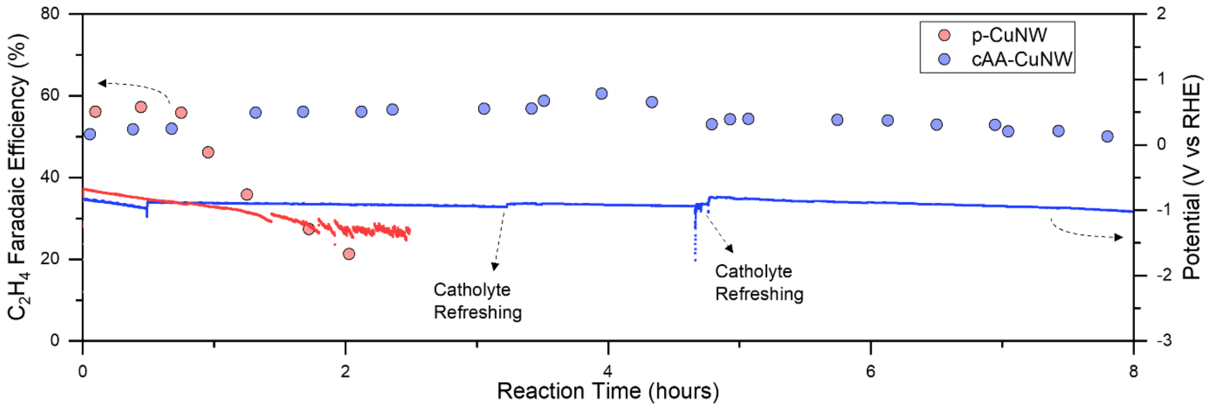

Supplementary Fig. 36 | Comparison of CO_2RR stability between p-CuNW and cAA-CuNW in flow cell electrolyzer with 1 M KOH electrolyte. CO_2RR of p-CuNW and cAA-CuNW proceeded at a total current density of 300 mA/cm^2 by chronopotentiometry.

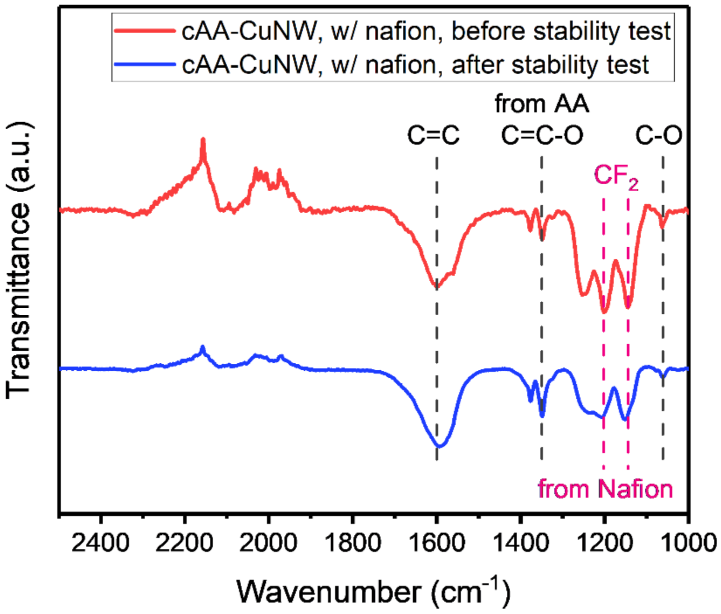

Supplementary Fig. 37 | FT-IR spectra of cAA-CuNW before and after CO_2RR stability test in the flow cell. When we compared the FT-IR peaks before and after CO_2RR of cAA-CuNW, the peaks for C=C stretching vibration, C=C−O asymmetric stretching vibration of the enol-hydroxyl group, and C−O vibration in the functional groups of AA were identical. The peaks at 1,207 and 1,153 cm^{-1} corresponding to asymmetric and symmetric CF_2 stretch mode in Nafion, respectively. FT-IR analysis was performed by FT-IR spectrometer (Nicolet Continuum, Thermo Scientific) with attenuated total reflection (ATR) accessory.

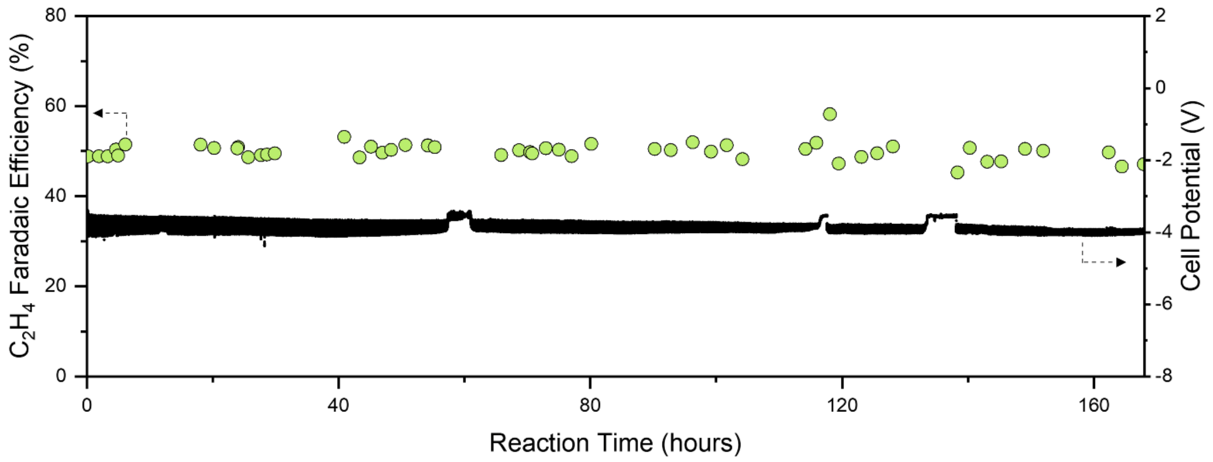

Supplementary Fig. 38 | Long-term C_2H_4 production of cAA-CuNW in a membrane electrode assembly (MEA) electrolyzer with 0.1 M $KHCO_3$ electrolyte. CO_2RR of cAA-CuNW proceeded at a total current density of 150 mA/cm^2 by chronopotentiometry. IrO_2 deposited Ti frit was used as an anode. The cathode and anode were separated by anion exchange membrane (Sustainion X37-50 RT, Dioxide Materials).

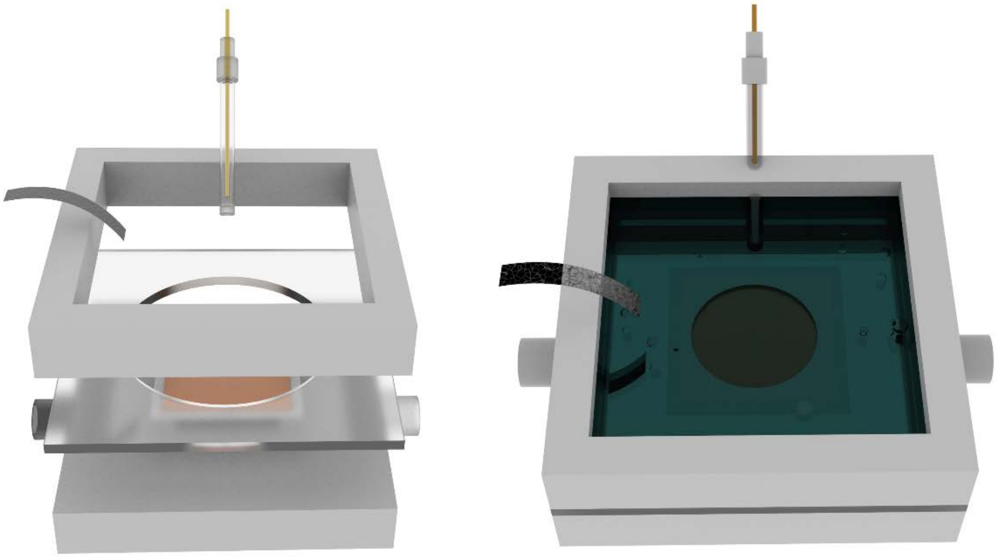

Supplementary Fig. 39 | Schematic of the flow cell reactor for in situ Raman spectroscopic analysis during the electrochemical CO_2RR. The immersion objective lens is placed through the exposed top to observe reaction intermediates on the sample, and CO_2 gas is supplied from the backside of the gas diffusion electrode.

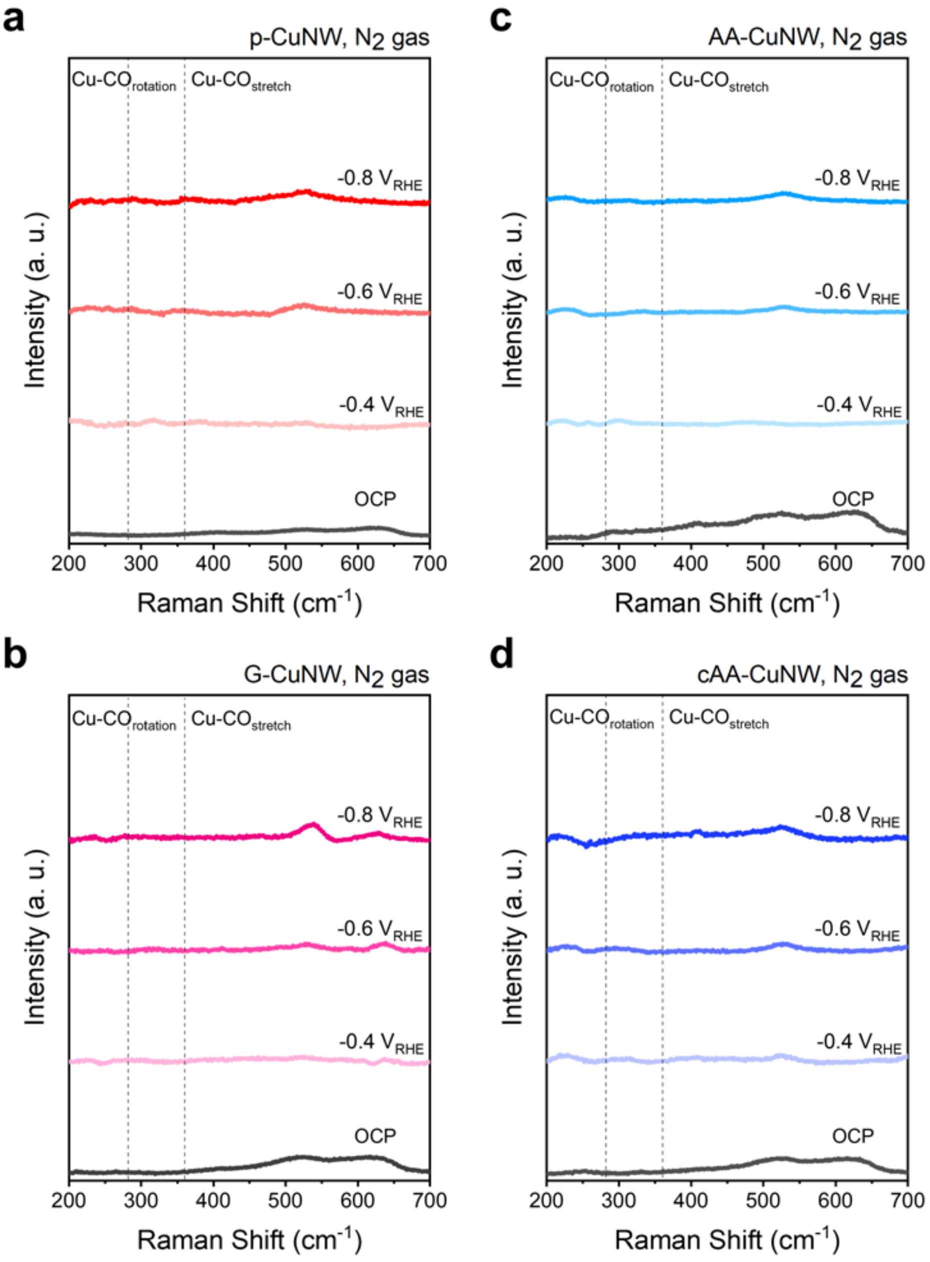

Supplementary Fig. 40 | Real-time observation of *CO bindings from CuNWs under N_2 gas. In situ Raman spectra of (**a**) p-CuNW, (**b**) G-CuNW, (**c**) AA-CuNW, and (**d**) cAA-CuNW obtained in the region of 200-700 cm^{-1} according to the applied potentials under N_2 gas.

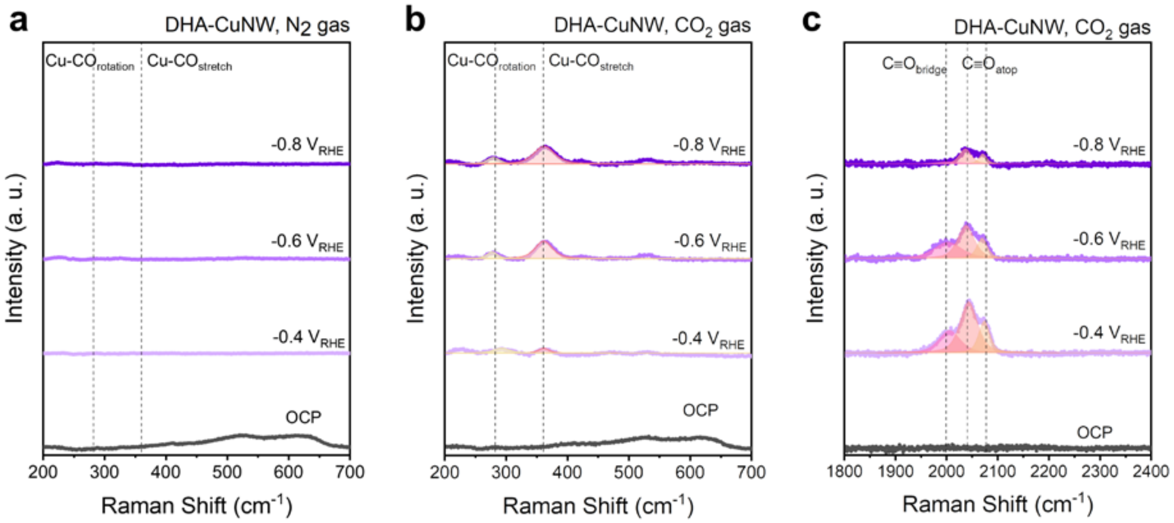

Supplementary Fig. 41 | Real-time observation of *CO bindings from DHA-CuNW. In situ Raman spectra of DHA-CuNW obtained in the region of 200-700 cm^{-1} according to the applied potentials under (**a**) N_2 and (**b**) CO_2 gas. **c** In situ Raman spectra of DHA-CuNW obtained in the region of 1,800-2,400 cm^{-1} according to the applied potentials under CO_2 gas.

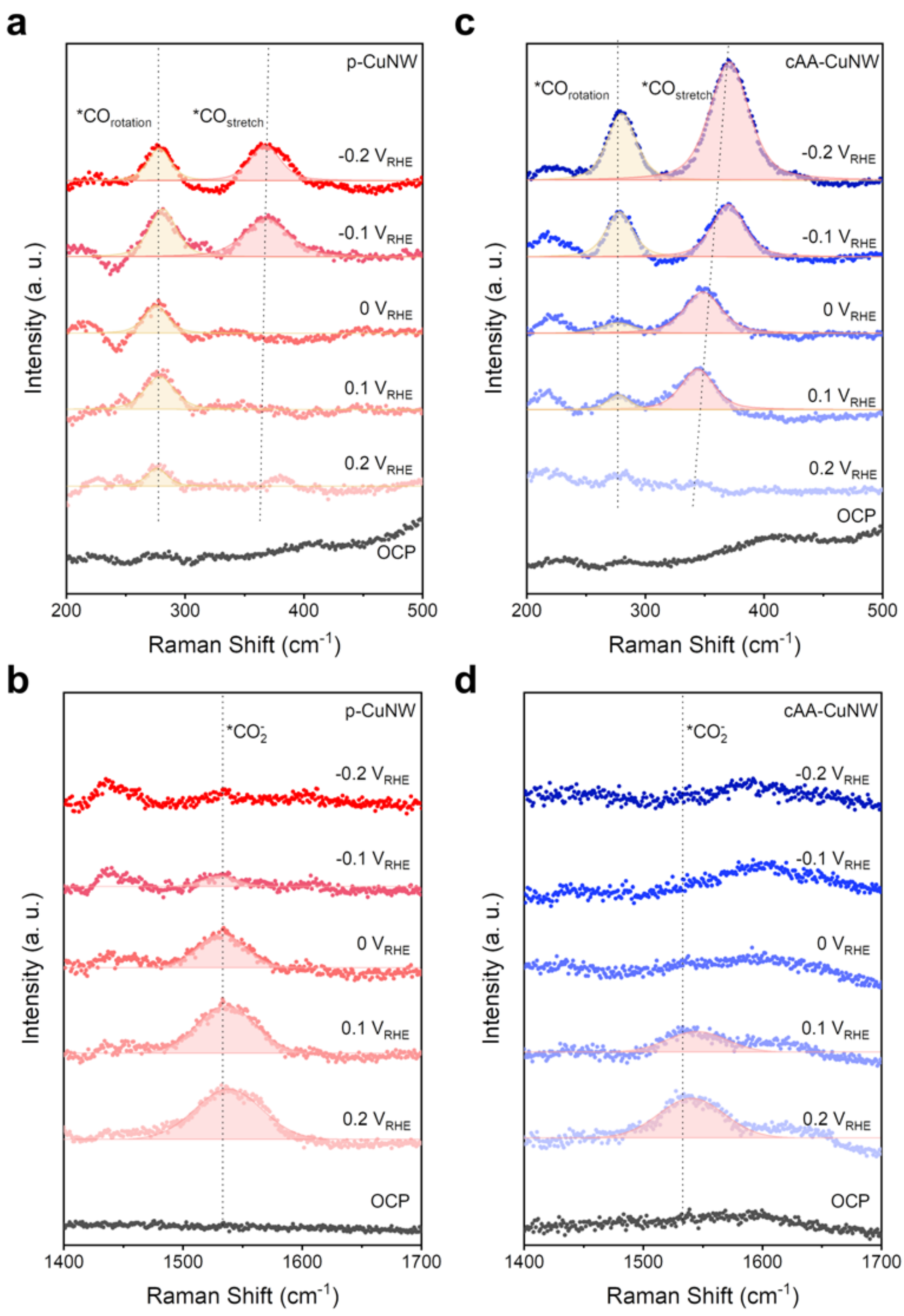

Supplementary Fig. 42│Monitoring *CO_2^- on CuNWs during CO_2RR. In situ Raman spectra of (**a**, **b**) p-CuNW and (**c**, **d**) cAA-CuNW obtained during CO_2RR according to the applied potentials in the region of 200-700 cm^{-1} (top) and 1,400-1,700 cm^{-1} (bottom). Cu-CO stretching peak for p-CuNW and cAA-CuNW appeared with the decrease of *CO_2^- peak intensity.

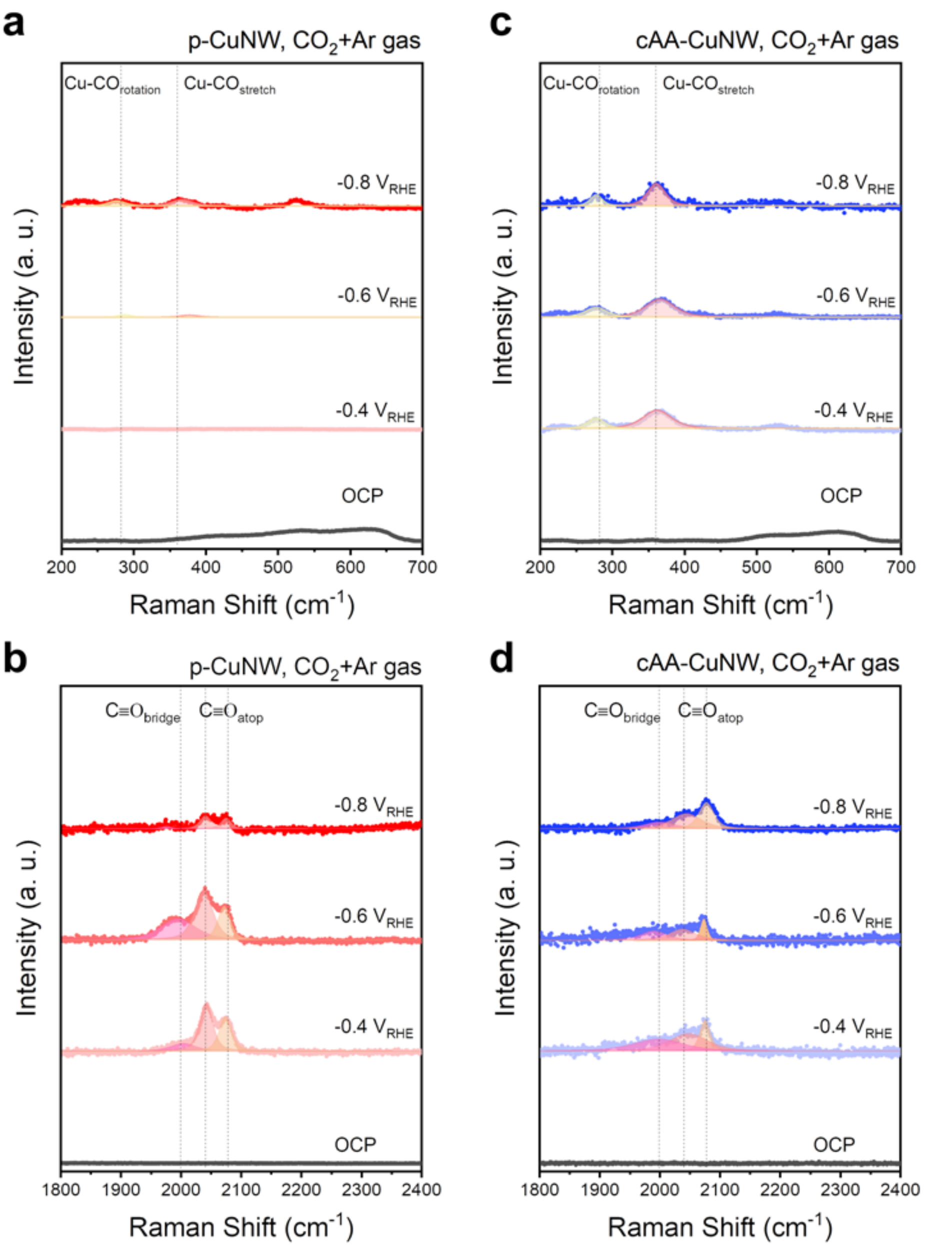

Supplementary Fig. 43 | Real-time observation of *CO bindings from CuNWs under CO_2+Ar mixed gas. In situ Raman spectra of (**a**, **b**) p-CuNW, and (**c**, **d**) cAA-CuNW obtained during CO_2RR according to the applied potentials in the region of 200-700 cm^{-1} (top) and 1,800-2,400 cm^{-1} (bottom).

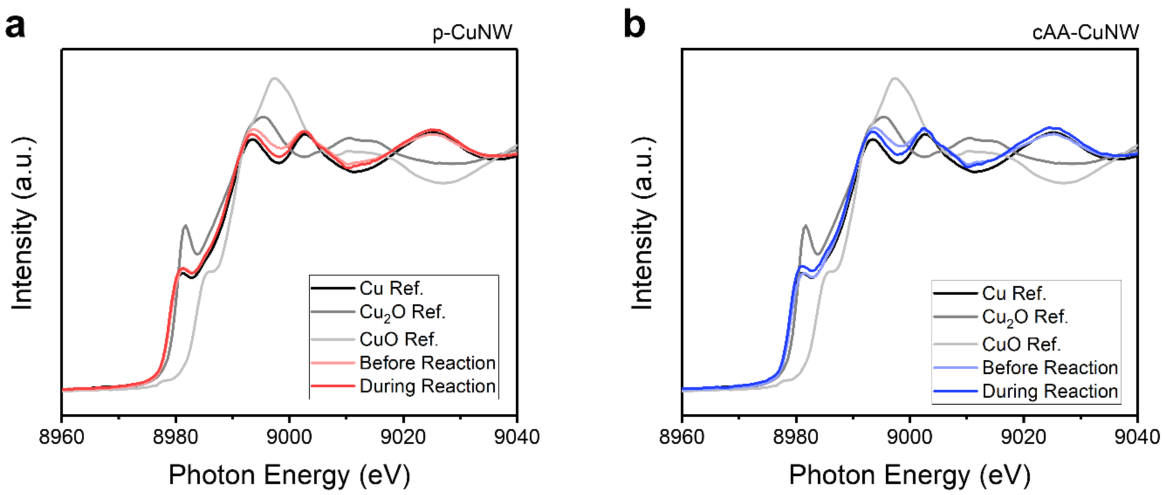

Supplementary Fig. 44 | Real-time oxidation state analysis of p-CuNW and cAA-CuNW during the CO2RR. Operando Cu K-edge XANES spectra obtained from (**a**) p-CuNW and (**b**) cAA-CuNW. The black and gray lines represent reference Cu and oxidized Cu spectra, respectively.

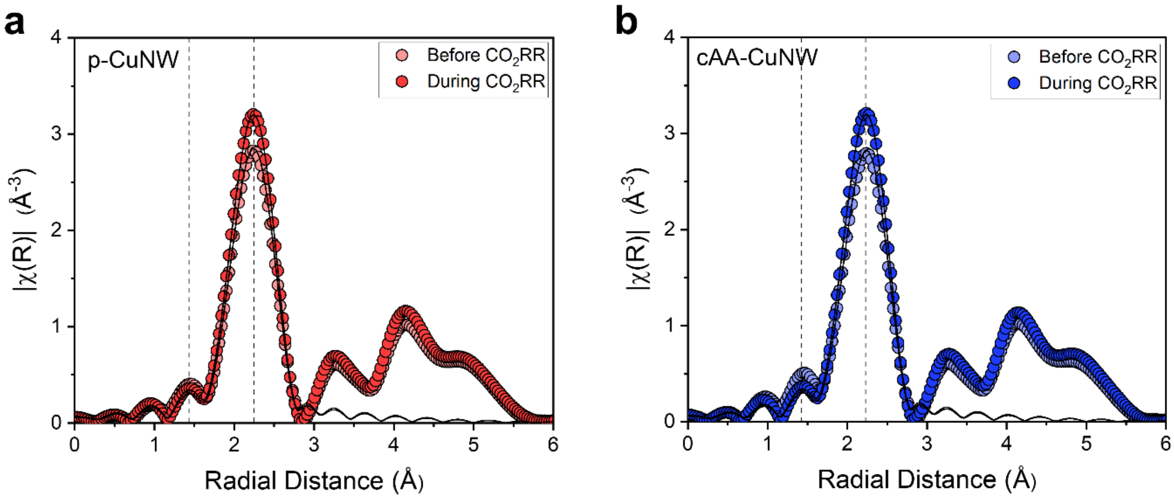

Supplementary Fig. 45 | Fitting curves of operando Cu K-edge EXAFS in R-space for p-CuNW and cAA-CuNW before and during the CO_2RR. Experimental curves (colored circles) and fitted curves (black line) of (**a**) p-CuNW and (**b**) cAA-CuNW.

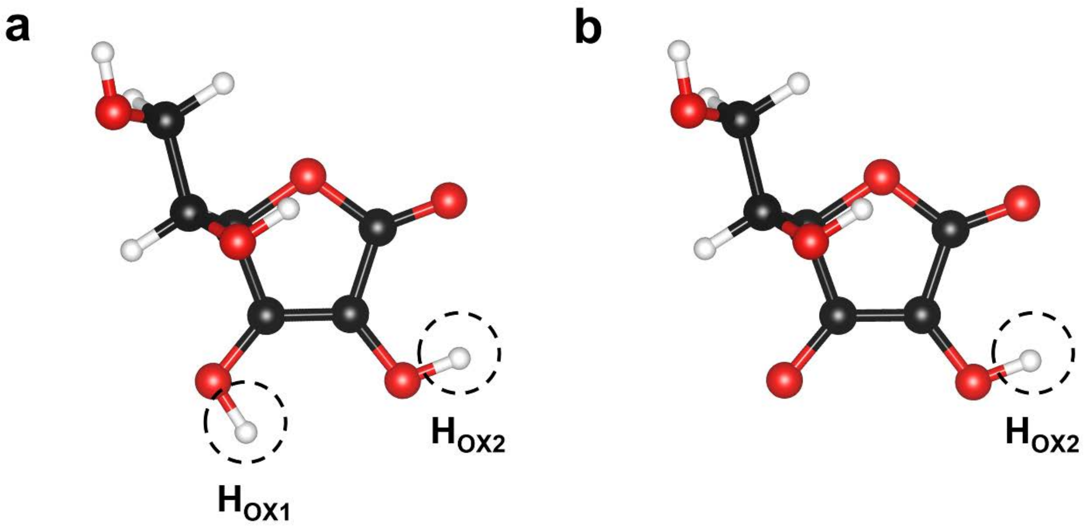

Supplementary Fig. 46 │ Atomic structures of (**a**) AA molecule and (**b**) ASC^- ion. Color code: black (C), white (H) and red (O). We considered the deprotonation of H_{OX1} for CO_2RR.[14]

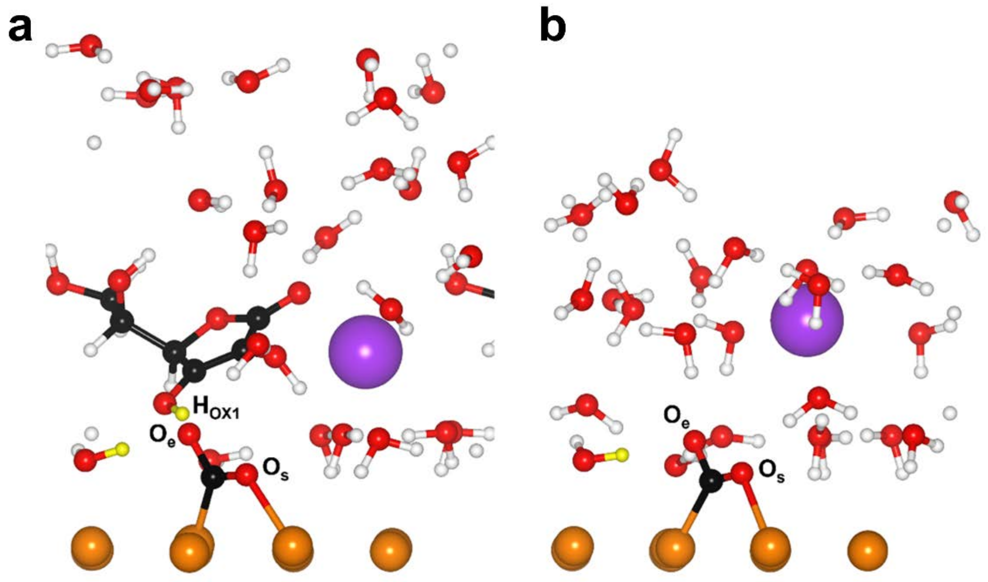

Supplementary Fig. 47 | ** Atomic structures of $*CO_2$ adsorption on (a**) AA/Cu (100) and (**b**) Cu (100). Color code: black (C), white (H), yellow (H), red (O), purple (K), and orange (Cu). Yellow H atoms are considered for the protonation during the CO_2RR.

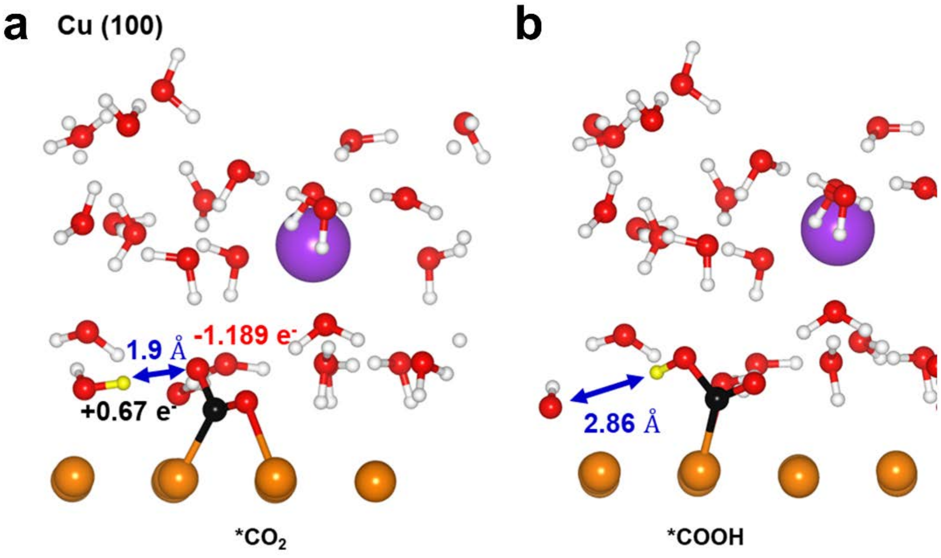

Supplementary Fig. 48 | Atomic structures of (**a**) the initial and (**b**) the final states of $^*CO_2$ protonation on Cu (100) ($* \, CO_2 + H_2O + e^- \rightarrow * \, COOH + OH^-$).

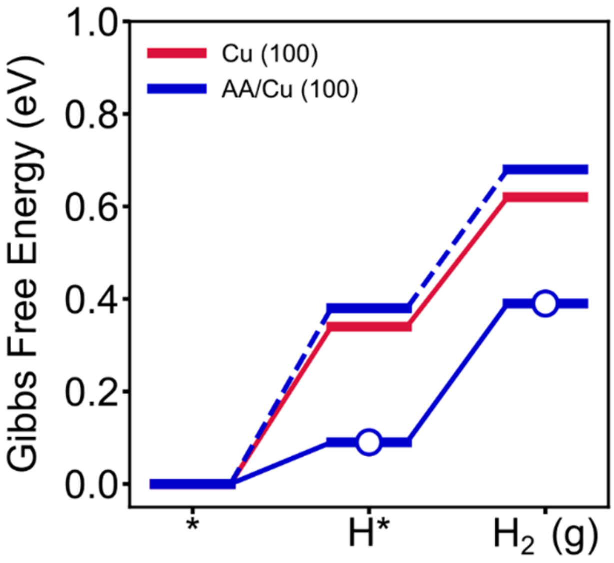

Supplementary Fig. 49 | The Gibbs free energy diagram of the HER. The reaction pathway involving the deprotonation of AA is highlighted with white circles, while H_2O is the proton source otherwise.

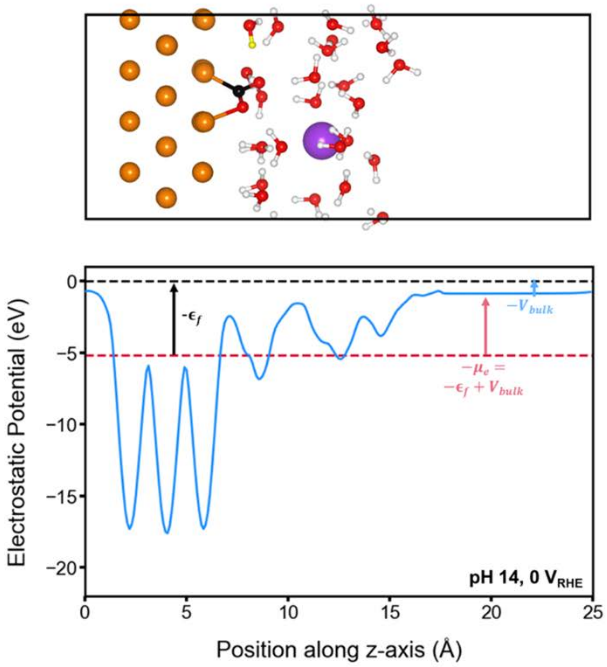

Supplementary Fig. 50 | An illustration of $*CO_2$ adsorption on Cu (100) and its electrostatic potential level along the z-direction at 0 V_{RHE}.

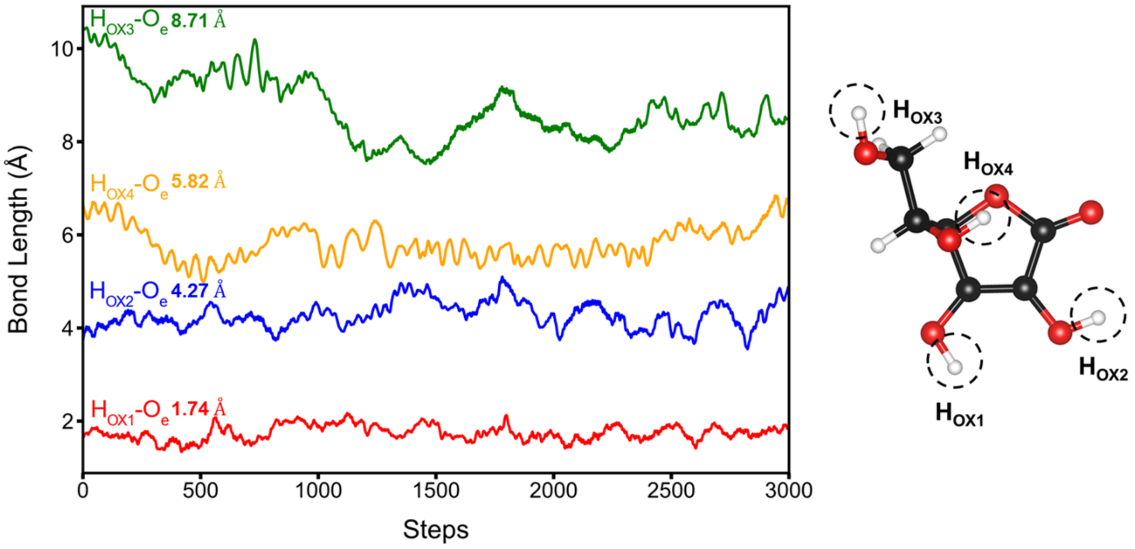

Supplementary Fig. 51 | The bond lengths between the hydrogen atoms in four distinct OH groups ($H_{OX1\sim4}$) in AA and the oxygen atom near the electrolyte (O_e) of $*CO_2$ measured during the last 3 ps of AIMD simulations.

**Supplementary Table 1 | **Comparison of various Cu-based CO_2RR catalysts for high-current C_2H_4 production.

Catalyst	Cell type	Electrolyte	J (mA/cm^2)	J_{C2H4} (mA/cm^2)	C_2H_4 FE (%)	V (vs RHE)	Ref.
cAA-CuNW	Flow cell	1 M KOH	888	539	60.7	-1.55	This work
		2 M KOH	805	453	56.3	-0.57	
S-HKUST-1	Flow cell	1 M KOH	400	229	57.2	-1.32	*Angew Chem. Int. Ed.* **61**, e202111700, (2022).[15]
Quasi-graphitic C shell on Cu	Flow cell	1 M KOH	400	284.4	71.1	-0.69	*Nat. Commun.* **12**, 3765, (2021).[16]
Nanoporous Cu	Flow cell	1 M KOH	653	252	38.6	-0.67	*Adv. Mater.* **30**, 1803111, (2018).[17]
Fluorinated Cu	Flow cell	0.75 M KOH	1,600	1040	65	-0.89	*Nat. Catal.* **3**, 478–487, (2020).[18]
Cu NPs + Nafion on Cu	Flow cell	7 M KOH	1,550	930	60	-3.23	*Science* **367**, 661–666, (2020).[19]
Cu(100) grown under CO_2	Flow cell	7 M KOH	580	388	67	-0.71	*Nat. Catal.* **3**, 98–106, (2020).[20]
Cu film on PTFE	Flow cell	3.5 M KOH + 5 M KI	720	473	66	-0.67	*Science* **360**, 783–787, (2018).[21]
Porous Cu	MEA	Pure Water	900	420	46.6	3.54	*Nat. Energy* **7**, 835-843, (2022).[22]
CAL-modified Cu	Slim, low-resistance flow cell	1 M H_3PO_4 + 3 M KCl	1,200	372	31	4.2	*Science* **372**, 1074-1078, (2021).[23]
CuO NS	Flow cell	1 M $KHCO_3$	700	231	33	.	*Nat. Commun.* **12**, 794, (2021).[24]
Cu-12	Flow cell	1 M $KHCO_3$	322	232	72	-0.83	*Nature* **577**, 509-513, (2020).[25]

Supplementary Table 2 | Cu–Cu atomic distance (R), coordination number (CN), and fitting parameters in operando EXAFS analysis of CuNW and cAA-CuNW.

Condition	Bonding	R (Å)	CN	σ^2 (Å^2)	ΔE_0 (eV)
p-CuNW before CO_2RR	Cu-Cu	2.54	10.269	0.0087	5.36
p-CuNW during CO_2RR	Cu-Cu	2.54	11.557	0.0087	5.29
cAA-CuNW before CO_2RR	Cu-Cu	2.54	10.266	0.0088	5.20
cAA-CuNW during CO_2RR	Cu-Cu	2.54	11.706	0.0089	5.07

Supplementary Table 3 │ The Bader charges of adsorbates on Cu (100) and AA/Cu (100). *OCCO are more reduced on AA/Cu (100) than on Cu (100).

	Cu (100)	AA/Cu (100)
**$*CO_2$**	$-1.16\ e^-$	$-1.07\ e^-$
***OCCO**	$-1.39\ e^-$	$-1.53\ e^-$

Supplementary Table 4 | Gibbs free energy correction values of gaseous molecules and adsorbates. For CO_2 and H_2, partial pressures of 101,325 Pa were set, respectively. All values of zero-point energy (ZPE), enthalpic ($\int C_p \, dT$), and entropic (-TΔS) contributions are given in eV.

	**ZPE**	$\int \boldsymbol{C_p \, dT}$	**-TΔS**
**CO_2 (g)**	0.304	0.098	-0.663
**H_2 (g)**	0.291	0.065	-0.403
**$*CO_2$**	0.283	0.101	-0.206
***COOH**	0.604	0.102	-0.209
***CO**	0.175	0.075	-0.132
***2CO**	0.358	0.151	-0.303
***OCCO**	0.366	0.135	-0.277
***H**	0.122	0.013	-0.019

Supplementary References

1. Garza, A. J., Bell, A. T. & Head-Gordon, M. Mechanism of CO_2 reduction at copper surfaces: pathways to C_2 products. *ACS Catal.* **8**, 1490-1499 (2018).
2. Li, Z. et al. Revisiting Reaction Kinetics of CO Electroreduction to C_{2+} Products in a Flow Electrolyzer. *Energy Fuels* **37**, 7904-7910 (2023).
3. Zhang, T. et al. Regulation of functional groups on graphene quantum dots directs selective CO_2 to CH_4 conversion. *Nat. Commun*. **12**, 5265 (2021).
4. Dou, L. et al. Solution-processed copper/reduced-graphene-oxide core/shell nanowire transparent conductors. *ACS Nano* **10**, 2600-2606 (2016).
5. Huang, S. et al. High-performance suspended particle devices based on copper-reduced graphene oxide core–shell nanowire electrodes. *Adv. Energy Mater*. **8**, 1703658 (2018).
6. Chua, C. K. & Pumera, M. C Chemical reduction of graphene oxide: A synthetic chemistry viewpoint. *Chem. Soc. Rev*. **43**, 291-312 (2014).
7. Agarwal, V. & Zetterlund, P. B. Strategies for reduction of graphene oxide–A comprehensive review. *Chem. Eng. J*. **405**, 127018 (2021).
8. Georgakilas, V. et al. Noncovalent functionalization of graphene and graphene oxide for energy materials, biosensing, catalytic, and biomedical applications. *Chem. Rev*. **116**, 5464-5519 (2016).
9. Yang, M., Hou, Y. & Kotov, N. A. Graphene-based multilayers: Critical evaluation of materials assembly techniques. *Nano Today* **7**, 430-447 (2012).
10. Griessl, S. et al. Self-assembled two-dimensional molecular host-guest architectures from trimesic acid. *Single Mol*. **3**, 25-31 (2002).
11. Wang, Y., Shi, Z. & Yin, J. Facile synthesis of soluble graphene via a green reduction of graphene oxide in tea solution and its biocomposites. *ACS Appl. Mater. Interfaces* **3**, 1127-1133 (2011).

12. Lin, Y. et al. Tunable CO_2 electroreduction to ethanol and ethylene with controllable interfacial wettability. *Nat. Commun.* **14**, 3575 (2023).

13. Cho, H.-H., Yang, H., Kang, D. J. & Kim, B. J. Surface engineering of graphene quantum dots and their applications as efficient surfactants. *ACS Appl. Mater. Interfaces* **7**, 8615-8621 (2015).

14. Shen, J. et al. Ascorbate oxidation by iron, copper and reactive oxygen species: review, model development, and derivation of key rate constants. *Sci. Rep.* **11**, 7417 (2021).

15. Wen, C. F. et al. Highly ethylene-selective electrocatalytic CO_2 reduction enabled by isolated Cu−S motifs in metal–organic framework based precatalysts. *Angew. Chem. Int. Ed.* **61**, e202111700 (2022).

16. Kim, J.-Y. et al. Quasi-graphitic carbon shell-induced Cu confinement promotes electrocatalytic CO_2 reduction toward C_{2+} products. *Nat. Commun.* **12**, 3765 (2021).

17. Lv, J.-J. et al. A highly porous copper electrocatalyst for carbon dioxide reduction. *Adv. Mater*. **30**, 1803111 (2018).

18. Ma, W. et al. Electrocatalytic reduction of CO_2 to ethylene and ethanol through hydrogen-assisted C–C coupling over fluorine-modified copper. *Nat. Catal*. **3**, 478–487 (2020).

19. García de Arquer, F. P. et al. CO_2 electrolysis to multicarbon products at activities greater than 1 A cm^{-2}. *Science* **367**, 661–666 (2020).

20. Wang, Y. et al. Catalyst synthesis under CO_2 electroreduction favours faceting and promotes renewable fuels electrosynthesis. *Nat. Catal*. **3**, 98–106 (2020).

21. Dinh, C.-T. et al. CO_2 electroreduction to ethylene via hydroxide-mediated copper catalysis at an abrupt interface. *Science* **360**, 783–787 (2018).

22. Li, W. et al. Bifunctional ionomers for efficient co-electrolysis of CO_2 and pure water towards ethylene production at industrial-scale current densities. *Nat. Energy* **7**, 835-

843 (2022).

23. Huang, J. E. et al. CO_2 electrolysis to multicarbon products in strong acid. *Science* **372**, 1074-1078 (2021).

24. Wang, X. et al. Morphology and mechanism of highly selective Cu(II) oxide nanosheet catalysts for carbon dioxide electroreduction. *Nat. Commun*. **12**, 794 (2021).

25. Li, F. et al. Molecular tuning of CO_2-to-ethylene conversion. *Nature* **577**, 509-513 (2020).
